# Synthesis and In Vitro Activity of Novel Melphalan Analogs in Hematological Malignancy Cells

**DOI:** 10.3390/ijms23031760

**Published:** 2022-02-03

**Authors:** Anastazja Poczta, Piotr Krzeczyński, Joanna Tobiasz, Aneta Rogalska, Arkadiusz Gajek, Agnieszka Marczak

**Affiliations:** 1 Department of Medical Biophysics, Institute of Biophysics, Faculty of Biology and Environmental Protection, University of Lodz, 141/143 Pomorska Street, 90-236 Lodz, Poland; aneta.rogalska@biol.uni.lodz.pl (A.R.); arkadiusz.gajek@biol.uni.lodz.pl (A.G.); agnieszka.marczak@biol.uni.lodz.pl (A.M.); 2 Department of Pharmacy, Cosmetic Chemistry and Biotechnology, Team of Chemistry, Łukasiewicz Research Network—Industrial Chemistry Institute, 8 Rydygiera Street, 01-793 Warsaw, Poland; piotr.krzeczynski@ichp.pl (P.K.); joanna.tobiasz@ichp.pl (J.T.)

**Keywords:** apoptosis assay, chemical modification, DNA damage, in silico study, leukemia cell lines, melphalan

## Abstract

Despite the continuous developments in pharmacology and the high therapeutic effect of new treatment options for patients with hematological malignancies, these diseases remain a major health issue. Our study aimed to synthesize, analyze in silico, and determine the biological properties of new melphalan derivatives. We obtained three methyl esters of melphalan having in their structures amidine moieties substituted with thiomorpholine (EM–T–MEL), indoline (EM–I–MEL), or 4-(4-morpholinyl) piperidine (EM–MORPIP–MEL). These have not yet been described in the literature. The in vitro anticancer properties of the analogs were determined against THP1, HL60, and RPMI8226 cells. Melphalan derivatives were evaluated for cytotoxicity (resazurin viability assay), genotoxicity (alkaline comet assay), and their ability to induce apoptosis (Hoechst33342/propidium iodide double staining method; phosphatidylserine translocation; and caspase 3/7, 8, and 9 activity measurements). Changes in mitochondrial membrane potential were examined using the specific fluorescence probe JC–1 (5,5′,6,6′-tetrachloro-1,1′,3,3′–tetraethylbenzimidazol carbocyanine). The EM–T–MEL derivative had the highest biological activity, showing higher cytotoxic and genotoxic properties than the parent drug. Moreover, it showed a high ability to induce apoptosis in the tested cancer cells. This compound also had a beneficial effect in peripheral blood mononuclear cells (PBMC). In conclusion, we verified and confirmed the hypothesis that chemical modifications of the melphalan structure improved its anticancer properties. The conducted study allowed the selection of the compound with the highest biological activity and provided a basis for chemical structure-biological activity analyses.

## 1. Introduction

Blood cancers are currently among the most serious medical problems. Recent data have shown that one in eight cancer cases worldwide come from blood cells, bone marrow, or the lymphatic system. Malignant hematopoiesis, such as leukemia and lymphomas, is divided into more than 100 subtypes with varying survival rates [1]. Multiple myeloma (MM) is characterized by the infiltration of monoclonal plasma cells into the bone marrow, which secretes monoclonal immunoglobulin that is found in the urine and/or blood. The accumulation of these immunoglobulins leads to organ dysfunction, which causes hypercalcemia, renal failure, anemia, or lytic bone changes [2,3,4].

Treatment of MM involves a combination of drugs with distinct mechanisms of action, including immunomodulating drugs, proteasome inhibitors, monoclonal antibodies, alkylating agents, and histone deacetylase inhibitors. Despite these numerous modern therapies, high doses of melphalan followed by autologous stem cell transplantation remain key in treating MM patients eligible for transplantation. Melphalan is a bifunctional alkylating agent. Each of the two bis-2-chloroethyl melphalan groups forms carbonium intermediates that cause alkylation by covalent bonding with nitrogen atoms in the DNA of the guanine molecule at the 7-position. This leads to cross-links between the two DNA strands, preventing transcription and DNA replication and inhibiting the cell cycle. By targeting highly proliferating cells, including malignant plasma cells, this drug causes cell death [4].

A major limitation of chemotherapy, including melphalan treatment, is the lack of drug selectivity, which has adverse effects on healthy tissues. High doses of anticancer drugs also weaken patients’ immunity by affecting the body’s biomolecular defense system [5]. The therapeutic activity of melphalan, despite the high remission of the disease, is limited by melphalan numerous side effects, such as cardiotoxicity, irreversible myelosuppression, anemia, numerous infections, and kidney failure. Therapy-related secondary primary malignancies (SPMs) in myeloma patients have been recognized as a consequence of treatment with alkylating agents [6]. Another issue is the emergence of multidrug resistance during antimyeloma therapy [7]. Because of these problems, finding new forms of therapy is crucial for improving the effectiveness of therapy. Modifications of melphalan’s structure to improve its anticancer properties have been studied for years. As early as the 1950s and 1960s, scientists worked with melphalan derivatives and studied their activity on cell lines and in animal models [8,9,10]. Today, this topic is still relevant, and various melphalan analogs are being evaluated that will be more effective than the parent drug [2,11,12]. Chemical modifications of the drug structure could serve as the basis for an improved chemotherapy system for MM patients. Chemical modification of the structure and determination of the relationship between the chemical structure and its biological activity may be the basis for developing derivatives with optimal structure that can be used as future anticancer drugs. Targeted design of anticancer drugs based on well-designed and -conducted basic research may in the future replace long and expensive in vivo tests, preceding the qualification of a new structure for further preclinical research through its candidate for a future drug.

Our previous studies [13] have shown that chemical modifications of melphalan are a promising way of enhancing the activity of this molecule compared to that of the parent drug. It was shown that esterification of the carboxyl group and replacement of the amino group with an amidine group containing a morpholine ring caused significant changes in cells. The modified structures exceeded the cytotoxic, genotoxic, and proapoptotic activity of the unmodified melphalan. Based on this knowledge, in the current research, we performed further chemical modifications of both the carbonyl and amino groups of melphalan to find derivatives with higher anticancer activity. As a result, we obtained methyl esters of melphalan containing amidine residues, including thiomorpholine (EM–T–MEL), indoline (EM–I–MEL), and 4-(4-morpholinyl) piperidine (EM–MORPIP–MEL). Our current research aimed to determine the cytotoxicity and genotoxicity of the new melphalan derivatives, as well as the ability of these derivatives to induce apoptosis and generate changes in the mitochondrial membrane potential. Such derivatives could potentially be therapeutically important.

## 2. Results

### 2.1. Chemistry

**Figure 1:**
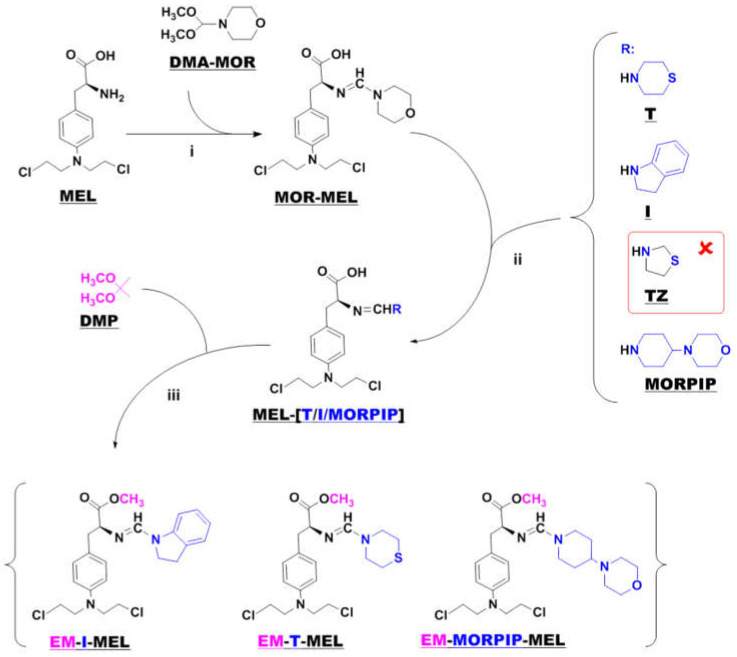
Synthesis of new melphalan derivatives. Conditions: (**i**): methanol, room temperature, 1 h; (**ii**): methanol, room temperature; (**iii**): 2,2–dimethoxypropane, concentrated hydrochloric acid, room temperature, 1 h.

Samples of melphalan derivatives used in biological research were obtained by three-stage chemical synthesis from commercially available substrates (Figure 1).

The first step in the synthesis involved the reaction of commercial melphalan (MEL) with a 50% molar excess of N-formylmorpholine dimethylacetal (DMA–MOR) in methanol at room temperature. The morpholine derivative of melphalan (MOR–MEL) obtained was the starting material for the further synthesis of all new derivatives.

MOR–MEL was then reacted with a 100% molar excess of the appropriate amine (thiomorpholine (T), indoline (I), or 4-(4-morpholinyl) piperidine (MORPIP)) in methanol at room temperature to obtain the appropriate amine derivative of melphalan (MEL–T, MEL–I, and MEL–MORPIP). Initially, we intended to obtain a thiazolidine derivative of melphalan in place of MEL–MORPIP, but during the experiments, it turned out that MOR–MEL did not react with thiazolidine (TZ). Attempts to change the reaction conditions and the synthesis method were unsuccessful. Therefore, it was decided to discontinue further work and replace the thiazolidine derivative with 4-(4-morpholinyl) piperidine.

In the last stage, the amine derivatives of melphalan (MEL–(T/I/MORPIP)) were esterified. Ester synthesis was carried out with 2,2-dimethoxypropane (DMP) in an acidic environment (approximately 10% addition of concentrated hydrochloric acid) to give the corresponding amine derivatives of melphalan methyl esters in the form of thick oils free of amines. All three amines were then converted to a hydrochloride form with a 5 M solution of hydrogen chloride gas in ethyl acetate.

Because of these structural changes, their molecules were much less polar than melphalan itself. This was confirmed by HPLC analysis (Figure S1), which showed the composition of the peaks of individual compounds. The analysis was performed on the reversed phases. The retention times of the tested derivatives ranged from 13,424 min to 20,531 min, while the retention time of melphalan under the same conditions was 12,151 min, which indicated the greater polarity of the melphalan molecule than of the described derivatives.

### 2.2. In Silico Analysis

To determine whether the test compounds belonged to the druglike group, the rules of Lipinski and the definition of Veber were used. According to the five Lipinski rules, a drug candidate should meet the following criteria: molecular weight (MW) ≤ 500 Da; lipophilicity described as logP ≤ 5; number of hydrogen bond acceptors (HBAs) ≤ 10; and number of hydrogen bond donors (HBDs) ≤ 5 [14]. The data obtained for the new test compounds are summarized in Table 1.

All the test compounds complied with Lipinski’s rules and had only one violation of Veber’s rules. The number of rotational bonds was slightly greater than the number specified in Veber’s rule. We also considered that none of the new compounds was a substrate for P-glycoprotein.

**Figure 2:**
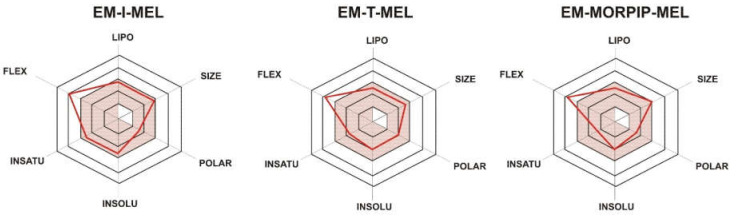
RADAR charts for the physicochemical properties of lipophilicity (LIPO), size, polarity (POLAR), solubility (INSOLU), saturation (INSATU), and flexibility (FLEX) determined for EM–I–MEL, EM–T–MEL, and EM–MORPIP–MEL.

To illustrate the distribution of the most important physicochemical properties in the body, we also prepared radar charts (Figure 2) from the SwissADME website, which consider six important physicochemical properties. The pink area represents the optimal range for each property: lipophilicity (LIPO), XLOGP3 between −0.7 and +5.0; size (SIZE), MW between 150 and 500 g/mol; polarity (POLAR), TPSA between 20 and 130 Å^2^; solubility (INSOLU), log S not higher than 6; saturation (INSATU), fraction of carbons in the sp^3^ hybridization not less than 0.25; and flexibility (FLEX), no more than nine rotatable bonds.

For all the compounds tested, five of the six properties analyzed were located in the pink area, which proved their optimality and compliance with the properties typical for compounds belonging to the group of druglike compounds. Based on these properties, it was also concluded that the test compounds were predicted not to be orally bioavailable because they were too flexible. Intravenous administration would be recommended.

### 2.3. Chemical Modifications of Melphalan Alter Its Cytotoxicity in Human Cells in In Vitro Study

**Figure 3:**
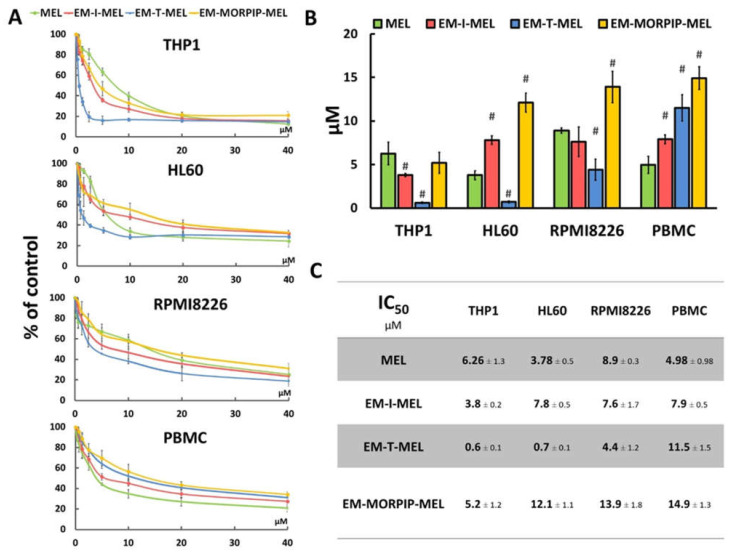
Chemical modifications of the melphalan molecule significantly increased cytotoxicity against leukemic cells and reduced cytotoxic activity against normal cells. (**A**) Concentration-dependent cytotoxic effects of MEL and its derivatives on HL60, THP1, RPMI8226, and PBMC growth measured by the resazurin assay. Cells without any treatment were used as controls and taken as 100%. (**B**) Graphical interpretation of IC_50_ values. (#) *p* < 0.05 statistically significant differences between samples incubated with melphalan and melphalan derivatives. (**C**) IC_50_ values for melphalan and melphalan derivatives against the cancer cell lines tested.

The cytotoxic activity of the compounds was assessed using a resazurin reduction assay after 48 h of incubation. The sensitivity of cells to the test compounds was determined using the IC_50_ parameter (Figure 3).

The cell lines tested showed different sensitivities to the parent compound and the obtained derivatives. The THP1 cells showed the highest sensitivity to the test analogs, while the RPMI8226 line had the lowest sensitivity. EM–I–MEL had higher (about 1.5 times) cytotoxicity than the parent drug in only the THP1 cells. In all cancer cell lines tested, the highest cytotoxicity was observed after incubation with EM–T–MEL. The highest activity of this compound (and the largest difference between the IC_50_ of melphalan and the IC_50_ of the derivative) was observed in human acute monocytic leukemia cells. The IC_50_ concentration of this derivative in this cell line was nearly 10 times lower than that of melphalan. In HL60 cells, the IC_50_ value of the EM–T–MEL derivative was approximately five times lower than that of the unmodified compound. Cells of the multiple myeloma line also showed high (two times higher than melphalan) cytotoxic activity after incubation with EM–T–MEL. At the same time, a decreased cytotoxic effect of EM–T–MEL was observed against peripheral blood mononuclear cells (PBMC). This analog was approximately 2.5 times less cytotoxic to PBMCs than melphalan. The cytotoxicity of EM–MORPIP–MEL was similar against THP1 and higher against HL60, RMPI8226, and PBMCs compared to the parent drug. Because of this, EM–MORPIP–MEL was eliminated from further analyses. One concentration per cell line was selected for testing of the next parameters: RPMI8226, 3 µM; HL60, 0.7 µM; THP1, 0.3 µM, which were the same as in previous studies evaluating the biological properties of melphalan analogs [13].

### 2.4. Tested Melphalan Analogs Caused DNA Fragmentation in Leukemic Cells

**Figure 4:**
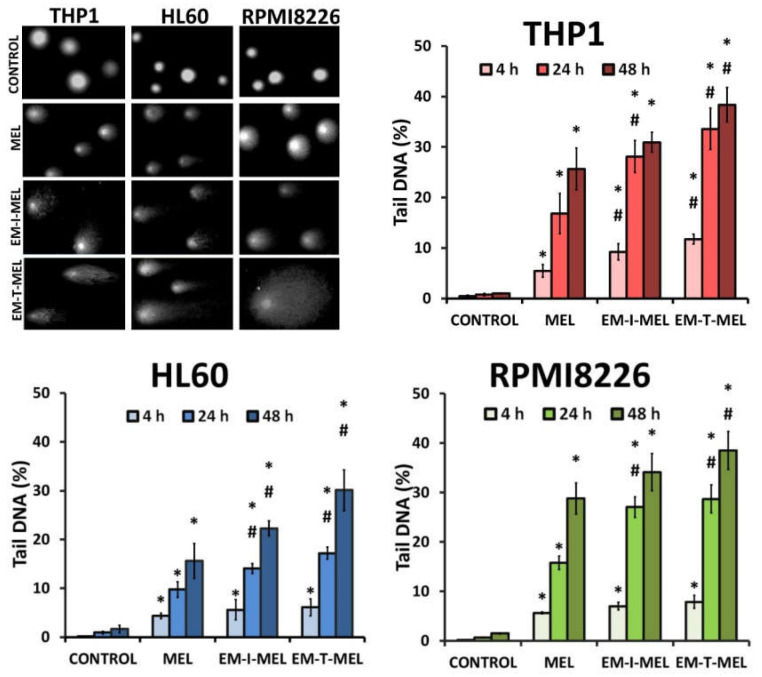
Melphalan analogs increased DNA damage (percentage of DNA in the comet tail) in THP1, HL60, and RPMI8226 cells after 4, 24, and 48 h of incubation. The characteristic tails of comets with damaged DNA are shown under a fluorescence microscope after electrophoresis and 4′,6-diamidino-2-phenylindole (DAPI) gel staining. All data are from three biological assays and are graphed as the mean ± SD. (*) Statistically significant differences compared to control cells, *p* < 0.05. (#) Statistically significant differences compared to MEL, *p* < 0.05.

The level of DNA damage in THP1, HL60, and RPMI8226 cells was analyzed by performing an alkaline version of the comet assay. Cells were incubated with melphalan and its derivatives (EM–I–MEL, EM–T–MEL) for 4, 24, and 48 h. Cells not treated with any compound were taken as control. Figure 4 shows DNA damage measured as a percentage of DNA in the comet tails of the cells tested. It was shown that melphalan and its derivatives induced DNA damage in the cells, and this increase was dependent on the incubation time. The most cytotoxic derivative, EM–T–MEL, also generated the highest level of DNA damage in all tested cells, mainly after 24 and 48 h of incubation. After as little as 4 h of incubation of the cells with the compounds, increased levels of DNA damage were observed. In THP1 cells at this time, unmodified melphalan caused 5.5% DNA strand breaks, while the EM–I–MEL derivative caused 9.2% DNA strand breaks. The EM–T–MEL derivative caused 11.7% DNA strand breaks. Statistically significant changes compared to melphalan were observed after 24 h incubation with EM–I–MEL and 24 and 48 h incubation with EM–T–MEL. Similar relationships were also observed in the HL60 cells. Treating HL60 cells for 48 h with the EM–T–MEL derivative (30.1%) was associated with twofold higher levels of DNA damage than was treating them with MEL (15.6%). The maximum level of myeloma cells with damaged DNA was observed after 48 h of treatment with derivatives EM–I–MEL (34.1%) and EM–T–MEL (38.5%).

### 2.5. Chemical Modifications Increase the Proapoptotic Properties of Melphalan: Using Double Staining to Determine the Mechanism of Leukemia Cell Death

#### 2.5.1. Hoechst 33342 and Propidium Iodide Double Staining

**Figure 5:**
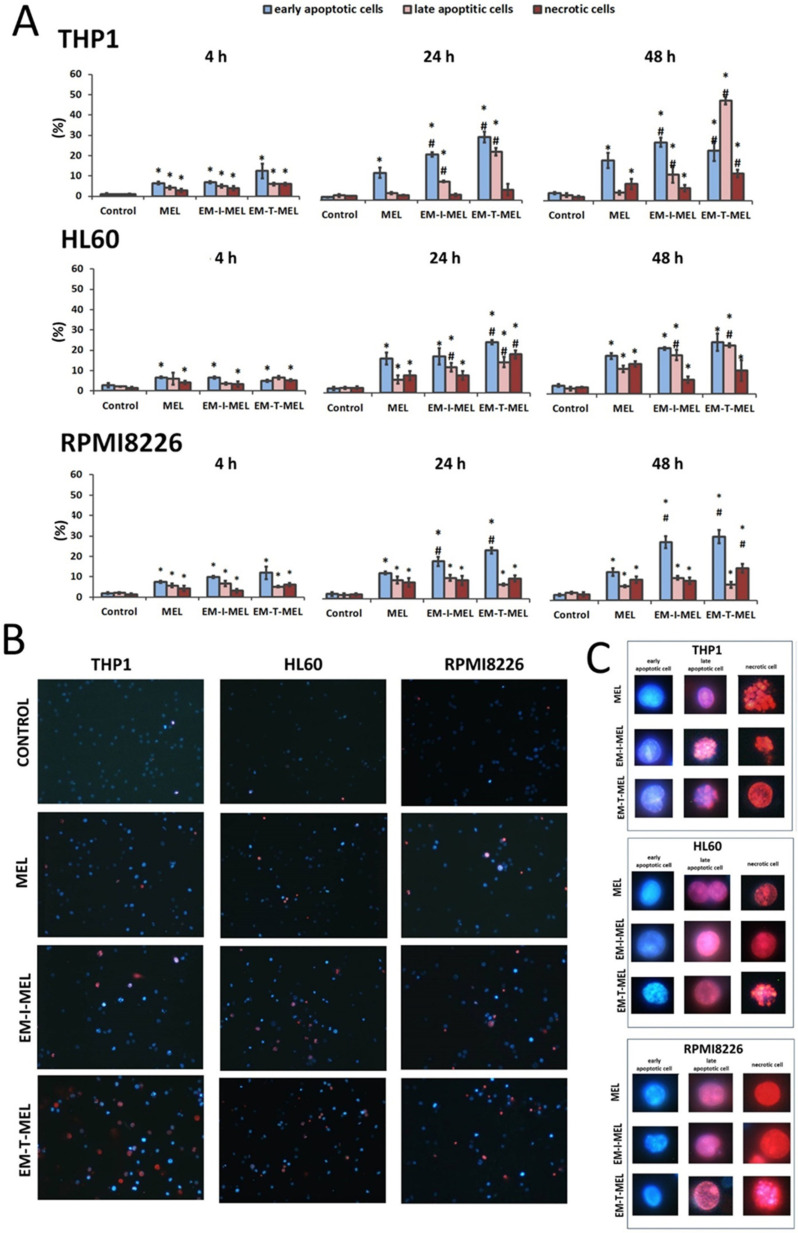
Novel melphalan analogs showed higher proapoptotic activity than the unmodified compound. (**A**) Fractions of apoptotic and necrotic cells after 4–48 h of incubation with MEL and test melphalan derivatives. Results are presented as the mean ± SD. (*) Statistically significant differences compared to control cells, *p* < 0.05. (#) Statistically significant differences compared to MEL, *p* < 0.05. (**B**) Determination of the fraction of apoptotic and necrotic cells after 48 h incubation with melphalan and melphalan derivatives. Cells were stained with the fluorescent dyes Hoechst 33342 and propidium iodide (PI) to determine the fraction of live cells (pale blue fluorescence), early-apoptotic (bright blue fluorescence), late-apoptotic (pink–violet fluorescence), and necrotic (red fluorescence) cells. (**C**) Morphological changes observed in the THP1, HL60, and RPMI8226 lines after 48 h of incubation with melphalan and the derivatives EM–I–MEL and EM–T–MEL. The cells were visualized under a fluorescence microscope (Olympus IX70).

The simultaneous use of two fluorescent dyes—propidium iodide (PI) and Hoechst 33342—enabled the identification of four populations of cells in the microscope image field: live, early-apoptotic, late-apoptotic, and necrotic cells. The experiment was carried out after 4, 24, and 48 h of incubation with the test compounds. Figure 5B,C shows the morphological changes, while the quantitative analysis of the live cell fraction and the early apoptotic, late apoptotic, and necrotic cells are shown in Figure 5A.

In the case of THP1 cells, after 24 h of incubation with the test compounds, a significant increase in the number of cells in the early stage of apoptosis was observed with EM–I–MEL (23% of the total population) and EM–T–MEL (31% of the total population) compared to MEL (13% of the total population). After a 4-h incubation, significant changes were observed only in relation to the control. The greatest changes were observed after 48 h incubation with the EM–T–MEL derivative. There was a significant increase in the pool of late apoptotic cells (49% of the total population) compared to control cells (2.5%) and cells treated with melphalan (4%). There was also a significant increase in the number of cells in the early stage of apoptosis (25%). It is also worth noting that treatment of THP1 cells resulted in the appearance of a fraction of necrotic cells. The necrotic cell content in the entire population was 13%. These changes were statistically significant relative to the control and parent drug. The EM–I–MEL derivative led mainly to an increase in the number of early apoptotic cells (29% of the total population) and late apoptotic cells (13%) in the longest incubation period with the compound. The 48 h incubation of THP1 cells with the parent drug was associated mainly with an increase in the pool of early apoptotic cells (20%).

The 24 and 48 h incubations of HL60 cells with MEL and MEL derivatives resulted in significant increases in the population of apoptotic and necrotic cells. After 24 h of incubation, the EM–I–MEL derivative initiated the appearance of about 31% of the apoptotic cell fraction (early apoptotic cells: 17% of the entire population; late apoptotic cells: 14%; necrotic cells: 8.5%). In the case of the EM–T–MEL derivative, an even greater percentage of apoptotic cells was recorded, approximately 40% (early-apoptotic cells: 25% of the total population; late-apoptotic cells: 15%), and 19% of the entire population was necrotic cells. The 48 h incubation of HL60 cells resulted in a significant increase in the fraction of late apoptotic cells for both the EM–I–MEL derivative and the EM–T–MEL derivative compared to the parent compound.

In the case of multiple myeloma cells, after 24 and 48 h of incubation with melphalan and the test analogs, a significant increase in the number of early-apoptotic cells was found. Furthermore, 48-h incubation with EM–I–MEL or EM–T–MEL derivatives resulted in the appearance of about 40% of the total population as apoptotic cells (early and late apoptosis). This increase was about two times higher than that in the cells treated with the parent drug. There was also a significant increase in the number of necrotic cells after incubation with the EM–T–MEL derivative (16% of the total population) compared to after incubation with MEL (10%).

#### 2.5.2. Annexin V-Fluorescein Isothiocyanate, and Propidium Iodide Double Staining

**Figure 6:**
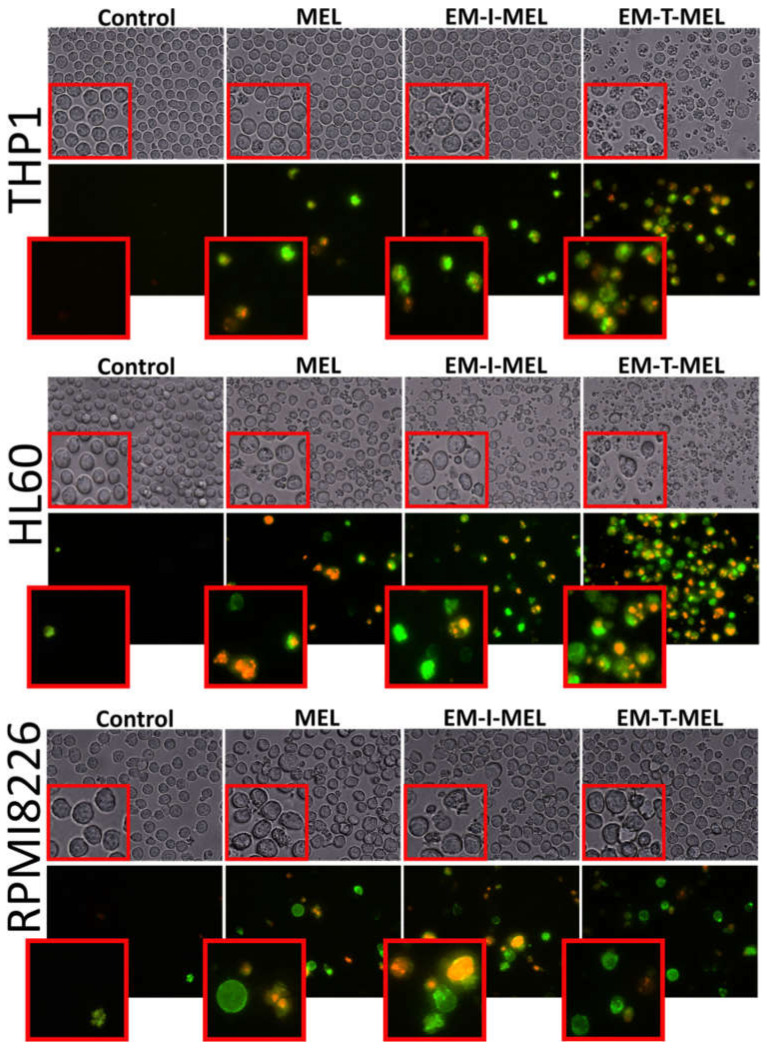
The investigated derivatives induced changes in cell membrane integrity and externalization of phosphatidylserine. We visualized THP1, HL60, and RPMI8226 cells by fluorescence microscopy (Olympus IX70, Japan) after 24 h of incubation with MEL and the test derivatives. The cells were stained with annexin V–fluorescein isothiocyanate (FITC) and PI. After incubation with MEL derivatives, mainly EM–T–MEL cells showed high green (derived from annexin V–FITC) and red fluorescence (derived from PI) indicative of exposed phosphatidylserine (PS) and damage to cell membrane integrity, characteristic features of apoptosis.

One marker of cell death by apoptosis is a disruption in the cell membrane asymmetry and externalization of phosphatidylserine. Therefore, we performed a qualitative assessment of the molecular events associated with apoptosis using the annexin V–fluorescein isothiocyanate (FITC) and PI double staining method (Figure 6). We also included bright-field observation images in order to visualize morphological changes after treatment with the investigated compounds. EM–T–MEL caused the most significant changes in single-cell morphology, such as shrinkage, apoptotic body formation, and cell fragmentation. At the same time, cells after incubation with the test compounds, mainly EM–T–MEL, showed high green (derived from annexin V–FITC) and red fluorescence (derived from propidium iodide), indicating phosphatidylserine translocation and damage to cell membrane integrity.

### 2.6. Melphalan Derivatives Activated Caspase-Dependent Apoptosis: Determining the Apoptotic Pathway in Cancer Cells

The anticancer properties of compounds are primarily due to their ability to induce apoptosis. We assayed the activity of the executive caspase 3/7 and initiator caspases 8 and 9, which are directly involved in the activation of this process. The final results are expressed as the percentage of activity of a specific cysteine protease, with the fluorescence value of the untreated control taken as 100%.

**Figure 7:**
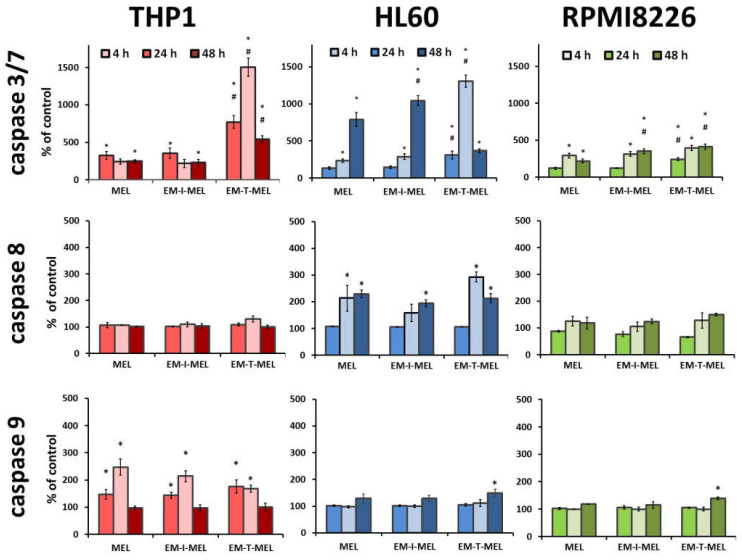
Melphalan derivatives activated executive caspase 3/7 and initiator caspases 8 and 9 in THP1, HL60, and RPMI8226 cells. Cells were incubated for 4, 24, and 48 h with MEL, EM–I–MEL, and EM–T–MEL. The final results are expressed as the percentage of activity of a specific cysteine protease, with the untreated control taken as 100%. All data are from three biological assays and are graphed as the mean ± SD. (*) Statistically significant differences compared to control cells, *p* < 0.05. (#) Statistically significant differences compared to MEL, *p* < 0.05.

In the case of THP1 cells, increased activity of mainly caspase 3/7 was observed (Figure 7). After a 24 h incubation with the EM–T–MEL (1506%) derivative, the increase in activity of this cysteine protease was about six times higher than that after incubation with the unmodified MEL (243%). Incubations for 4 h and 24 h of THP1 cells with all test compounds resulted in activation of caspase 9. However, the changes were statistically significant only relative to the control. HL60 cells were also highly sensitive to caspase 3/7 activation. Significant changes in the activity of this caspase were observed mainly after 24 h of incubation with the EM–T–MEL derivative (1307%) compared to the parent drug (233%). After 48 h incubation, statistically significant changes were also observed for the derivative EM–I–MEL (1044%). The increased activity of this caspase was preceded mainly by the activation of the initiating caspase 8 after incubation with all test compounds. The changes were statistically significant only compared to the control. RPMI8226 cells induced caspase 3/7 mainly after 24 and 48 h of incubation. In contrast, the most cytotoxic EM–T–MEL derivative caused an increase in the activity of this caspase after only 4 h of incubation. In contrast, this line was the least sensitive to the activation of this cysteine protease. There was also no increase in the activity of the analyzed initiating caspases (8 and 9) during the tested incubation times with the compounds.

### 2.7. The New Derivatives of Melphalan Caused Loss of Mitochondrial Membrane Potential

**Figure 8:**
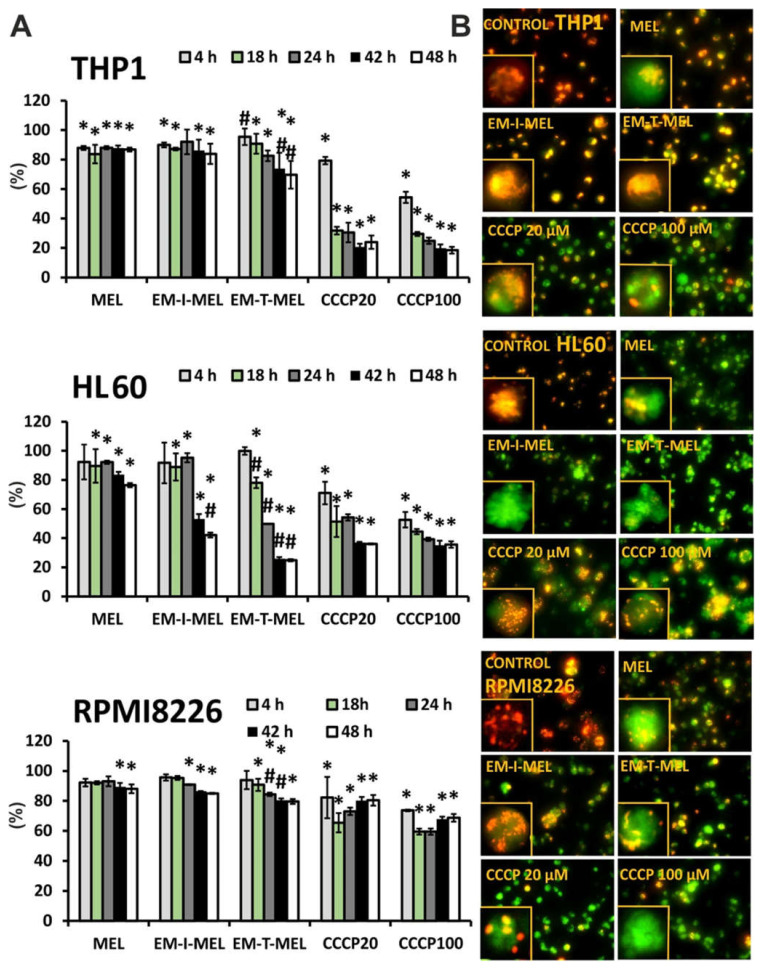
New melphalan derivatives induced a decrease in mitochondrial membrane potential. (**A**) The fluorescence ratio of JC–1 dimers/JC–1 monomers in the control was assumed to be 100%. The cells were stained with the fluorescence probe JC–1 prior to 4–48 h of incubation with MEL, EM–I–MEL, EM–T–MEL, or carbonyl cyanide *m*–chlorophenyl hydrazone (CCCP) (20, 100 µM). All data are from three biological assays and are graphed as the mean ± SD. (*) Statistically significant differences compared to control cells, *p* < 0.05. (#) Statistically significant differences compared to MEL, *p* < 0.05. (**B**) Fluorescent microscopy images of HL60, THP1, and RPMI8226 cells stained with JC–1 probe after 48 h. The cells were viewed under an inverted fluorescence microscope (Olympus IX70, Japan, 400× magnification).

The disruption of mitochondrial integrity is a key event in apoptosis. Depolarization of the mitochondrial membrane is often associated with apoptosis by the release of proapoptotic proteins, such as cytochrome c, from the mitochondria and the formation of a proapoptotic complex. The impact of MEL, EM–I–MEL, and EM–T–MEL on the mitochondrial membrane potential (ΔΨm) in HL60, RPMI8226, and THP1 cells was assessed using fluorometric analysis after staining with the fluorescent dye 5,5′,6,6′-tetrachloro-1,1′,3,3′-tetraethylbenzimidazolcarbocyanine (JC–1) (Figure 8). Red fluorescence of JC–1 dimers (high mitochondrial potential) was observed in control cells. Treatment with new compounds dramatically increased the green fluorescence of JC–1 monomers, indicating decreases in the mitochondrial membrane potential. All compounds induced time-dependent changes. In all the cell lines studied, the greatest changes in the mitochondrial potential took place after EM–T–MEL treatment.

The highest green fluorescence intensity was observed in HL60 cells treated with the EM–T–MEL for 24–48 h (ΔΨm dropped to 24.8% vs. control). Already after 18 h of treatment, a statistically significant difference was noticed between the action of the derivative and that of MEL. EM–I–MEL also led to a significant reduction in ΔΨm (to 42%) compared to MEL (to 76%) after 48 h of treatment. In the acute monocytic leukemia cells, strong green fluorescence of JC–1 monomers was observed after treatment with both new melphalan derivatives. Furthermore, in this line, the derivative EM–T–MEL significantly decreased the potential against melphalan. The RPMI8226 line turned out to be the least sensitive to the activity of the test compounds regarding ΔΨm changes. Interestingly, this line was also sensitive to the action of EM–I–MEL and EM–T–MEL, but after a longer incubation time. Chlorophenylhydrazone (CCCP), an uncoupler of oxidative phosphorylation, was used as a positive control for the depolarization of ΔΨm.

## 3. Discussion

Blood cancers remain a major health issue despite the high therapeutic efficacy of new treatment options and continuous pharmacological developments. In a previous study [13], we verified and confirmed the hypothesis that chemical modifications to melphalan increased its anticancer properties. We showed that esterification of the carboxyl group was necessary to improve the effectiveness of melphalan. In addition, the additional replacement of the amino group with an amidine group containing two heteroatoms (O and N) in the structure significantly increased the properties of the drug.

The subject of the present research was the analysis of three new melphalan derivatives with increased cytotoxic activity in human neoplastic cells that have not yet been described in the literature. New chemical modifications to old, well-known drugs require a balance between size and lipophilicity to improve cytotoxicity in leukemia therapy. Two of the most effective melphalan modifications were the addition of a morpholine group in our previous work [13] and a thiomorpholine group in the current work. Morpholine and its analogues represent effective heterocycles because of their conformational and physicochemical properties. The oxygen atom in this ring increases binding affinity by participating in donor–acceptor-type interactions with the corresponding receptor. Moreover, the oxygen atom reduces the basicity of the nitrogen atom of the morpholine ring by its electronegative effect [15,16]. Two heteroatoms in the amidine group at the opposite position provide flexible conformation to the ring. Thanks to this construction, this group can participate in lipophilic−hydrophilic interactions. Morpholine modulates properties of the overall structure, as the presence of a weak basic nitrogen at the opposite position of the oxygen atom enhances solubility [17]. Morpholine derivatives can act as antidepressants, analgesics, antitumor agents, antioxidants, antibiotics, or antifungals as well as serving in the treatment of central nervous system disorders [18]. The analog with the rest of the thiomorpholine is part of the previously adopted strategy, because thiomorpholine has two heteroatoms (N and S) in its structure. We attempted to synthesize and research a system that has not been tested so far, i.e., the use of a bicyclic structure consisting of two rings in the amidine group (analog with the rest of the indoline) and the use of a bicyclic structure additionally containing two nitrogen atoms and an oxygen atom. In addition to the described modifications to the amino group, the resulting derivatives were converted to methyl esters according to the established observations that ester analogs are more cytotoxic than the free carboxyl group analogs. We assessed the cytotoxic, genotoxic, and proapoptotic properties against multiple myeloma (RPMI8226) and two types of myeloid leukemia (acute promyelocytic leukemia (HL60) and acute monocytic leukemia (THP1)) to determine the biological activity of the new analogs.

Investigations of the pharmacological properties of new molecular chemical entities (NMEs) or major drug discovery candidates have accelerated in recent years because of the high failure rate of drug candidates in clinical trials. The incorporation of in vitro studies prior to the absorption, distribution, metabolism, elimination, and toxicity (ADMET) study is a promising strategy to improve research on future drug candidates. Drug toxicity is one of the most relevant properties and the most unpredictable characteristic of a drug, as it can be species- and organ-specific. The toxicity of a compound to different cell types can be evaluated using easily available and widely applied in vitro cytotoxicity assays. The advantage of these assays is that they facilitate the process of screening large chemical libraries [19]. Our study used well-known and recommended screening methods to assess the toxicity and intracellular mechanisms associated with the cytotoxic effects of compounds with anticancer potential.

A significant clinical problem in patients with advanced cancer is the high toxicity of chemotherapy. Melphalan is responsible for forming covalent cross-bonds between DNA strands, preventing transcription and replication, leading to cell death. The number of intrastrand cross-links formed is correlated with both the in vitro cytotoxicity of the drug and the patient’s response to treatment [20]. We assessed the cytotoxic activity of the test compounds against neoplastic cells and normal PBMCs. The cytotoxic effect of MEL and its derivatives was dose dependent. The IC_50_ parameter, i.e., the concentration necessary to inhibit the biological process in vitro by 50%, was determined. The EM–T–MEL derivative showed the highest cytotoxicity against neoplastic cells, with the IC_50_ value being about tenfold (THP1), fivefold (HL60), and twofold (RPMI8226) lower than the unmodified compound. Simultaneously, this compound was significantly less (2.5-fold) cytotoxic than melphalan to PBMCs. The EM–I–MEL analog was also more cytotoxic than MEL, exhibiting significantly lower IC_50_ values in the THP1 cancer cell model. Since the cytotoxic activity of the EM–MORPIP–MEL derivative was comparable or less than that of the parent drug, it was eliminated from further analyses. These results confirmed the finding from our previous studies [13] that chemical modifications involving esterification and conversion of an amino group to an amidine group with two heteroatoms exhibit high cytotoxic properties against cancer cells and low cytotoxicity to PBMCs. Esterification and modification of the amino group significantly modify the properties of the compound. Thus, when explaining the different cytotoxic activities of the analyzed compounds, the size, structure, and ring geometry of the attached amine should be taken into account. There have also been reports in the literature that esterification can increase bioavailability and cellular penetration [21,22,23,24]. In addition, other in vitro studies have shown that amidine analogs of melphalan reduce the number of estrogen receptor-positive and estrogen receptor-free breast cancer cells [25,26].

Melphalan is mainly transported to the cells via large neutral amino acid transporter 1 (LAT1) [7,27]. It has been shown that the level of LAT1 expression in tumor tissues is significantly higher than in surrounding healthy tissues [28]. Nonsolid tumors exhibit altered LAT1 expression: LAT1 acts as an activating antigen in T cells, and T cell leukemia results in higher levels of LAT1 expression compared to levels in normal activated T cells [27]. We observed higher differences in the sensitivity of normal and leukemic cells in the new derivatives than in melphalan. Leukemic cells require large amounts of nutrients and amino acids for rapid growth and continuous proliferation. The new derivatives, because of the reduced polarity of the molecule in comparison to melphalan, may have wider delivery strategies. EM–T–MEL, EM–I–MEL, or EM–MORPIP–MEL can be transported to cancer cells by other membrane receptors that are overexpressed in leukemia cancer cells: MCT-1, -2, and -4, for lactate transport; OAT-1, for small, hydrophilic organic anion transport; OCTN-1, FLIPT-1, and OCT-6, for organic cation transport; and OATP1B1 and OATP1B3, for large, hydrophobic anion transport [29]. P2X7R, an ATP-gated ion channel, is widespread in cancer cells and overexpressed in AML. Recent studies have indicated that P2X7R-activated macropore opening was shown to enhance the intracellular uptake of drugs including chemotherapeutics such as doxorubicin. P2X7R-activated macropore opening was therefore proposed as tumor cell-specific drug delivery system [30].

Melphalan, as an alkylating drug, is responsible for an increase in the level of the DNA double break marker (γH2AX), induces the phosphorylation of checkpoint kinase 1 (CHK-1) and checkpoint kinase 2 (CHK-2) in MM cells (RPMI8226 and MM1.S) [31], and leads to apoptosis through the formation of intra- and interstitial DNA cross-bonds [32]. Confirming the genotoxic properties of melphalan in selected models of neoplastic cells (THP1, HL60, RPMI8226) and determining whether the new derivatives cause an increase in the level of DNA damage have become further goals of our research. Both the parent drug and the derivatives induced DNA damage in a time-dependent manner. The EM–T–MEL derivative resulted in the highest level of DNA damage in all tested cells, which correlated with the cytotoxicity analysis.

Understanding the mechanism of death of a cancer cell exposed to the test compounds was one of the main research problems of this study. Apoptosis is an important goal of chemotherapy as a process that is strictly regulated and controlled by specific biochemical processes requiring the expression of many different genes. Abnormal apoptosis is a key factor in resistance to chemotherapy. The ability of drugs to induce apoptosis is considered an important criterion in assessing their therapeutic efficacy. Apoptosis is the preferred type of cell death, as it is a noninflammatory physiological process. Unlike necrosis, apoptosis is characterized by specific morphological and biochemical changes, including cell shrinkage, fragmentation of the cell nucleus, chromatin condensation, disturbance in cell membrane asymmetry, and the formation of small vesicles (ApoBD) [33,34]. One early marker of apoptosis is a change in cell membrane asymmetry and phosphatidylserine translocation. The tested melphalan analogs induced apoptosis in the tumor cells. Based on the results obtained, the test compounds were characterized by different degrees of influence on the leukemic cells and multiple myeloma, manifested by changes in cell morphology. The greatest change was observed after 24 h of incubation with the test compounds, mainly the EM–T–MEL derivative, which correlated with the analysis of the cytotoxicity of the test analogs. Examination of the morphological changes after Hoechst 33342/PI and annexin V–FITC/PI staining suggested that the EM–T–MEL derivative triggered apoptosis-specific markers more clearly than the unmodified MEL in all cell lines tested.

Another aspect of our current study focused on the biochemical changes occurring during apoptosis induced by the melphalan analogs. Melphalan and other DNA cross-linkers increase mitochondrial stress in HeLa and HCT cells and have been shown to be responsible for increasing the cytotoxicity of melphalan [35,36]. Research showed that melphalan induced apoptosis by mediating the mitochondrial permeability transition pore (mPTP) in cervical and colorectal cancer cells [36]. The involvement of mitochondria-dependent mechanisms is critical to the execution of intrinsic apoptotic pathways and is also required for executing the extrinsic apoptotic program. In HL60 cells, ΔΨm collapsed after only 4 h of MEL treatment. Treatment of HL60 cells with a mitochondrial uncoupling agent CCCP (50 µM for 1 h) caused a remarkable drop in ΔΨm [37]. Our data showed that following treatment with melphalan derivatives in cancer cell lines, the mitochondrial membrane potential dropped, especially at longer incubation times of 24–48 h. The changes were larger than those induced by the unmodified parent compound. The new melphalan derivatives, EM–I–MEL and EM–T–MEL, significantly disrupted the potential of the mitochondrial membrane, especially in the promyelocytic cell line. It has been shown that melphalan promotes rapid fragmentation of the mitochondrial network in a time-dependent manner and consequently the early activation of apoptosis.

Comprehension of the mechanisms by which cytotoxic drugs inhibit cancer cell proliferation and induce apoptosis is important for optimizing therapeutic efficacy. Programmed cell death (PCD) is an active, energy-dependent process that requires the activation of many genes and can proceed in various ways involving different cell organelles. Caspases—intracellular enzymes belonging to a large family of serine proteases—are involved in the process of apoptotic cell death. These enzymes are involved both at the initiation stage (initiating caspases include caspases 2, 8, 9, and 10) and at the final stage of apoptosis, the latter of which is referred to as the effector phase (effector caspases include caspases 3, 6, and 7). Caspase 9 is involved mainly in the intrinsic pathway of apoptosis, where mitochondria play a major role in apoptotic signaling and regulation of cell death processes. Caspase 8 is involved mainly in the intrinsic pathway mediated by receptor proteins [34,38]. Incubation with MEL and the test analogs resulted in activation of the effector caspase 3/7. We observed differences in sensitivity of the test analogs toward the tested cancer cells. The most sensitive cell lines were HL60 and THP1, while the least sensitive was the multiple myeloma line. The EM–T–MEL derivative most potently induced caspase 3/7 activation in all cell lines tested. In the case of THP1 and RPMI8226 cells, activation of this cysteine protease occurred after only 4 h of incubation with this analog. Activation of caspase 3/7 was preceded by the initiation of caspase 9 (mainly in THP1 cells) or caspase 8 (mainly in HL60 cells). The intrinsic pathway can be activated in the presence of intracellular signals or in response to the activation of death receptors on the plasma membrane. The intrinsic pathway is triggered by signals such as DNA damage leading to an imbalance between proapoptotic and antiapoptotic proteins in mitochondria and destabilization of mitochondrial membrane potential. In the case of RPMI8226 cells, no increase in the activity of caspase 8 or caspase 9 was observed. The low level of activation of these initiating caspases may suggest activation of a mechanism of cell death other than apoptosis. Mitotic catastrophe is a pathway of cell death caused mainly by microtubule-stabilizing or -destabilizing factors and DNA damage. It is triggered by mitotic failure due to dysfunctional cell cycle checkpoints and the development of aneuploid cells [39]. Mitotic failure occurs in a p53-independent manner and involves the activation of caspase 2 followed by the release of cytochrome c, activation of caspase 3, chromatin condensation and permeabilization of the mitochondrial membrane, and the subsequent formation [40,41,42] of mononucleated giant cells (MONGC) or multinucleated giant cells (MNOC) [43]. Mitotic catastrophe activation by melphalan was observed in multiple myeloma cells [44]. This may suggest activation of this death pathway in RPMI8226 cells after incubation with the melphalan analogs analyzed. Understanding the detailed mechanism of multiple myeloma cell death will be the goal of further research on new melphalan analogs.

## 4. Materials and Methods

### 4.1. General

Fine chemicals and solvents for synthesizing the analogs were purchased from commercial vendors and used without further purification.

Reactions were monitored by the TLC method. Analyses were performed on aluminum plates precoated with silica gel (Merck 60 F_254_, 0.25 mm). To develop the chromatogram, the following solvent system was used: methylene chloride/methanol/formic acid/water (82:15:2:1 *v*/*v*/*v*/*v*). Plates were visualized under UV light at 254 and 366 nm.

The chemical purity of MEL, MOR–MEL, and the products obtained in the subsequent stages of synthesis was tested by HPLC. The analyses were performed in a typical Waters system consisting of an autosampler, two pumps, a degasser, and a photodiode detector. The stationary phase was a Chromolith Performance RP18e column at 30 °C, and the mobile phase was a gradient of 0.1% trifluoroacetic acid (TFA) in water and 0.1% TFA in acetonitrile. The flow rate of the mobile phase was 1 ml/min. Relative retention time (RRT) is presented with reference to melphalan (see Figure S1).

Melting point measurements were carried out on an automatic Mettler Toledo MP70 system. For each compound, four capillary measurements were made in parallel, and the results were averaged.

Optical rotation measurements were performed on a Perkin Elmer 341 polarimeter at a wavelength of 589 nm in methanol at 20 °C using a standard 1 ml cuvette.

The IR spectra were recorded on a NICOLET 380 FTIR spectrometer with a high-pressure ATR attachment equipped with a germanium crystal. The characteristic bands were approximately 1690 cm^−1^ (>C=N–amidine) and approximately 1740 cm^−1^ (>C=O) (see Figures S2–S4)

^1^H NMR, ^13^C NMR, DEPT, COZY, HMBC, and HSQC spectra were measured using the Bruker AVANCE III HD 500MHz instrument at 500 MHz (^1^H) and 125 MHz (^13^C) at 25 °C for solutions in DMSO–*d*_6_ (see Figures S5–S10).

High-resolution mass spectrometry (HRMS) measurements were performed using a Synapt G2–Si mass spectrometer (Waters) equipped with an ESI source and quadrupole-time-of-flight mass analyzer. The mass spectrometer was operated in the positive ion detection mode. The optimized source parameters were: capillary voltage 3.2 kV, cone voltage 30 V, source temperature 110 °C, desolvation gas (nitrogen) flow rate 600 dm^3^/h, temperature 350 °C, and nebulizer gas pressure 6.5 bar. To ensure accurate mass measurements, data were collected in the centroid mode. The mass was corrected during acquisition using leucine enkephalin solution as an external reference (Lock-Spray™), which generated a reference ion at m/z 556.2771 Da ([M+H]^+^) in the positive ESI mode. The results of the measurements were processed using the MassLynx 4.1 software (Waters) incorporated with the instrument (see Figures S11–S16).

### 4.2. Synthesis

#### 4.2.1. 2-(Morpholinmethylideneamino)-3-[4-[bis(2-chloroethyl) amino] phenyl] Propanoic Acid (MOR–MEL)

Synthetic procedure and analytical data of this compound (under the name 2-(tetrahydro-1,4-oxazynomethylideneamino)-3-[4-[bis(2-chloroethyl) amino] phenyl] propanoic acid) come from: Ireana Oszczapowicz, Małgorzata Łukawska, Joanna Tobiasz, Anna Porębska, Agnieszka Owoc. Nowe pochodne melfalanu, sposób ich wytwarzania, zawierający je środek farmaceutyczny oraz zastosowanie medyczne. PL220880B1, p. 9, example 3. (available in polish language version only).

The reaction was carried out in an argon atmosphere. First, 305 mg (1 mmol) of melphalan was dissolved in 30 mL of methanol. Then, 195 mg (1.2 mmol) of *N*–(dimethoxymethyl)morpholine was added to this solution, and the mixture was stirred until the substrate disappeared (30 min at room temperature, TLC control). Then, the mixture was evaporated to dryness, and 4 ml methanol was added, followed by 35 ml of diethyl ether. The precipitated product was then filtered, washed with diethyl ether, and dried under vacuum. The expected product was obtained after recrystallization from the methanol/diethyl ether mixture and had the following characteristics. Mass: 310 mg (0.77 mmol). Yield: 77%. Off–white crystalline powder. 97.0% (HPLC). RRT (in ref. to MEL): 1.154, 1.201IR (cm^−1^): 1697 (>C=N–). ^1^H NMR (300 MHz, DMSO–*d*_6_, δ_ppm_): 9.30 (s, 1H, COOH), 7.74 (s, 1H, –N=CH–N–), 7.06 (d, *J* = 8.4 Hz, 2H, C_aryl_H_5/5′_), 6.71 (d, *J* = 8.4 Hz, 2H, C_aryl_H_6/6′_), 3.90 (m, 1H, –C_chiral_H–), 3.77 (m, 2H, –N_morph_–CH_2_–CH_2_–O–), 3.70 (s, 4H, Cl–CH_2_–CH_2_–N), 3.28 (m, 2H, –N_morph_–CH_2_–CH_2_–O–), 3.15 (dd, *J* = 3.9 Hz, *J* = 13.2 Hz, 1H, –CH_A_H_B_–CH(N=)–CO_2_H), 2.78 (dd, *J* = 10.8 Hz, 1H, –CH_A_H_B_–C_chiral_H–). Elemental analysis: calc. C—53.85%, H—6.28%, N—10.47%; found: C—53.65%, H—6.16%, N—10.27%.

New Melphalan Derivatives—General Procedure:

First, 1.61 g (4.0 mmol) of MOR–MEL was dissolved in 240 ml of methanol. Then, 8.0 mmol of the appropriate amine was added to this solution, and the mixture was stirred 18–22 h (depending on the amine) at room temperature (TLC control). Next, the mixture was evaporated to dryness, obtaining the crude product as an oil that was then purified by preparative HPLC, leading to the amine derivative of melphalan (oil, purity ca. 90% HPLC).

Then, 1 mmol (418–485 mg) of the melphalan amine derivative, 10 mL DMP, and 1 mL concentrated HCl were stirred for one hour at room temperature. The mixture was evaporated to dryness, and the resulting oil was purified by column chromatography to obtain the appropriate methyl ester with a purity of over 95% (HPLC). The free amine was then dissolved in 5 mL 4 M HCl_(g)_/ethyl acetate to obtain the hydrochloride that precipitated from the solution. The solid was filtered off, washed with cold ethyl acetate, and dried under vacuum at 30 °C to obtain the final product.

The following compounds were synthesized in this manner:

#### 4.2.2. 2-(Indolinmethylideneamino)-3-[4-[bis(2-chloroethyl) amino] phenyl] Propanoic Acid Methyl Ester Hydrochloride (EM–I–MEL):

Off-white crystalline powder. Yield: 25% (not optimized, calculated in relation to MOR–MEL). Purity: 98.80% (HPLC). RRT: 1.690. Mp.: decomposition below the melting point. [α]_d_^20^: −37.94° (c = 1, MeOH). IR (cm^−1^): >C=O (1743), >C=N– (1682). ^1^H NMR (500 MHz, 25 °C, DMSO–*d*_6_, δ_ppm_): OCH_3_ (3.72, s, 3H), C_chiral_H (4.65, bs, 1H), CH_A_H_B_C_chiral_H (3.18, dd, 1H), CH_A_H_B_C_chiral_H (3.02, dd, 1H), C5_arom_H+C5′_arom_H (7.11–7.13, d, 2H,), C6_arom_H+C6′_arom_H (6.65, d, 2H), N–CH_2_–CH_2_–Cl (3.62, bs, 4H), N–CH_2_–CH_2_–Cl (3.60, s, 4H), –N=CH– (8.81, bs, 1H), C2_ind_H (4.05, bs, 1H), C3_ind_H (3.26, bs, 1H), C4_ind_H+C6_ind_H (7,35, bs, 2H), C5_ind_H (7.26, bs, 1H), C7_ind_H (7.11–7.13, d, 1H,). ^13^C NMR (125 MHz, 25 °C, DMSO–*d*_6_, δ_ppm_): OCH_3_ (52.51), >C=O (170.4), C3 (36.40), C4 (124.10), C5/C5′ (130.50), C6/C6′ (111.80), C7 (145.15), N–CH_2_–CH_2_–Cl (51.96), N–CH_2_–CH_2_–Cl (40.96), C2_ind_ (48.07), C3_ind_ (27.24), C3a_ind_ (132.50), C4_ind_ (126.05), C5_ind_ (127.65), C6_ind_ (111.11), C7a_ind_ (149.98). HRMS: calc. for C_23_H_28_N_3_O_2_Cl_2_: 448.156; found: 448.155.

#### 4.2.3. 2-(Thiomorpholinmethylideneamino)-3-[4-[bis(2-chloroethyl) amino] phenyl] Propanoic Acid Methyl Ester Hydrochloride (EM–T–MEL)

Off–white crystalline powder. Yield: 30% (not optimized, calculated in relation to MOR–MEL). Purity: 96.27% (HPLC). RRT: 1.482. Mp.: decomposition below the melting point. [α]_d_^20^: –78.57° (c = 1, MeOH). IR (cm^−1^): >C=O (1748), >C=N– (1703). ^1^H NMR (500 MHz, 25 °C, DMSO–*d*_6_, δ_ppm_): OCH_3_ (3.72, s, 3H), C_chiral_H (4.37, bs, 1H), CH_A_H_B_C_chiral_H (3.14, dd, 1H), CH_A_H_B_C_chiral_H (2.90, dd, 1H), C5_arom_H+C5′_arom_H (7.09, d, 2H), C6_arom_H+C6′_arom_H (6.71, d, 2H), N–CH_2_–CH_2_–Cl (3.71, s, 4H), –N=CH– (7.83, bs, 1H), NCH_2_CH_2_S (3.61, bs, 4H), NCH_2_CH_2_S (2.62 and 2.35, 2 × bs (3:1), 4H). ^13^C NMR (125 MHz, 25 °C, DMSO–*d*_6_, δ_ppm_): OCH_3_ (52.49), >C=O (170.40), C2 (61.45), C3 (36.25), C4 (124.10), C5/C5′ (130.64), C6/C6′ (111.80), C7 (145.19), N–CH_2_–CH_2_–Cl (52.04), N–CH_2_–CH_2_–Cl (41.09), –N=CH– (155.13), NCH_2_CH_2_S (53.90, 47.30), NCH_2_CH_2_S (27.40, 25.80). HRMS: calc. for C_19_H_28_N_3_O_2_Cl_2_: 432.128; found: 432.127.

#### 4.2.4. 2-[[4-(Piperidin-4-yl)-morpholine] methylideneamino]-3-[4-[bis(2-chloroethyl) amino] phenyl] Propanoic Acid Methyl Ester Hydrochloride (EM–MORPIP–MEL)

Off–white crystalline powder. Yield: 41% (not optimized, calculated in relation to MOR–MEL). Purity: 97.85% (HPLC). RRT: 1.105. Mp.: decomposition below the melting point. [α]_d_^20^: –74.39° (c = 1, MeOH). IR (cm^−1^): >C=O (1744), >C=N– (1696). ^1^H NMR (500 MHz, 25 °C, DMSO–*d*_6_, δ_ppm_, conformation changes in both morpholine and piperidine rings caused the multiplication of some signals): OCH_3_ (3.71, s, 3H), C_chiral_H (4.43 and 4.37, bs, 1H), CH_2_C_chiral_H (3.07, bs, 2H), C5_arom_H+C5′_arom_H (7.08, t, 2H), C6_arom_H+C6′_arom_H (6.82, 6.72, 2d, 2H), N–CH_2_–CH_2_–Cl (3.70, bs, 2H), N–CH_2_–CH_2_–Cl (3.72, bs, 2H), –N=CH– (8.11, 7.92, 2d, 1H), N_pip_CH_A_H_B_ (4.53, 3.15, 3.80, 3.35, bs, 4H), N_pip_CH_2_CH_A_H_B_ (2.37, 2.33, 2.28, 1.84, bs, 4H), N_pip_CH_2_CH_2_CH (3.45, bs, 1H), NCH_A_H_B_CH_2_O (3.40, 3.05, 2bs, 4H), NCH_2_CH_2_O (3.86, bs, 4H). ^13^C NMR (125 MHz, 25 °C, DMSO–*d*_6_, δ_ppm_): OCH_3_ (52.70, 52.64), >C=O (170.29, 170.00), C2 (61.42, 60.68), C3 (35.90, 35.81), C4 (123.91, 123.85), C5/C5′ (130.53, 130.43), C6/C6′ (112.25, 111.97), C7 (145.32, 145.27), N–CH_2_–CH_2_–Cl (52.11, 52.05), N–CH_2_–CH_2_–Cl (41.31, 41.16), –N=CH– (155.08, 154.64), N_pip_CH_2_ (49.80, 49.75, 42.95, 42.83), N_pip_CH_2_CH_2_ (26.05, 25.91, 24.89, 24.57), N_pip_CH_2_CH_2_CH (60.59, 60.55), NCH_2_CH_2_O (48.42, 48.36, 48 23, 48.06), NCH_2_CH_2_O (63.10). HRMS: calc. for C_24_H_37_N_4_O_3_Cl_2_: 499.224; found: 499.224.

### 4.3. In Silico Analysis

A QSAR analysis was performed to see whether the new compounds met the criteria for druglike compounds. To provide a global pharmacokinetics profile of the test molecules, we used the freely accessible SwissADME web tool (http://www.swissadme.ch/, accessed on 5 December 2021) according to rules described in [45].

### 4.4. Cell Culture

Myeloma cancer cells (RPMI8226 (ATCC^®^ CCL–155™)), promyelocytic leukemia cells (HL60 (ATCC^®^ CCL–240™)), and acute monocytic leukemia cells (THP1 (ATCC^®^ TIB–202™)) were obtained from the American Type Culture Collection (ATCC, Rockville, MD, USA). PBMCs were isolated from buffy coat purchased from the Central Blood Bank (Lodz, Poland). PBMCs were isolated with Histopaque 1077 (Sigma Aldrich, St. Louis, MO, USA) by density gradient centrifugation at 300× *g* for 30 min at 22 °C. The final concentration of lymphocytes was estimated by trypan blue (0.4%, Sigma Aldrich) exclusion assay. All investigated cells were suspended in RPMI 1640 medium supplemented with 1% phytohemaglutinin (only in PBMC growth medium), 10% fetal bovine serum, penicillin (10 U/mL), and streptomycin (50 μg/mL) in standard conditions: 37 °C, 100% humidity, and an atmosphere of 5% CO_2_ and 95% air. Cell viability was systematically controlled using trypan blue (0.4%, Sigma). In all experiments, cells in the logarithmic phase of growth were used when their viability was above 95%.

### 4.5. Cytotoxicity Assay

The cytotoxic properties of melphalan (MEL) and its new derivatives (EM–I–MEL, EM–T–MEL, and EM–MORPIP–MEL) were investigated with the use of resazurin sodium after 48 h of incubation with the test compounds. Living cells were metabolically active and had the ability to reduce resazurin via mitochondrial reductase to a highly fluorescent dye, resorufin. The amount of product formed was directly proportional to the number of viable cells, while the intensity of the color produced was a quantitative measure of cell survival.

Cells were grown in 96-well black plates at 1.5 × 10^4^ cells/well and incubated with various concentrations of test compounds at 37 °C. After 48 h of incubation for each of the wells, resazurin solution (10 µg/mL, final concentration) was added, and the samples were incubated for 90 min. Fluorescence measurement was performed at ~530 nm excitation and ~590 nm emission using a Fluoroskan Ascent FL plate reader (Labsystems, Stockholm, Sweden).

### 4.6. Determination of Apoptotic and Necrotic Cell Fractions by Double Staining with a Mixture of the Fluorochromes Propidium Iodide and Hoechst 33342

PI is negatively charged and penetrates only cells with damaged cell membranes, which allows the identification of necrotic cells or cells in the late stages of apoptosis. Hoechst 33342, on the other hand, freely penetrates the intact membrane of living and early-apoptotic cells, thus enabling their identification. As a result of the dye penetrating the intact biological membranes, the DNA of the cell nucleus is stained a light blue color. The dye fluorescence intensity is related to the degree of DNA packing, which allows, on the basis of the intensity of fluorescence of the fluorochrome in the cell nucleus, distinguishing strongly fluorescent apoptotic cells containing highly condensed chromatin from weakly fluorescing living cells containing looser chromatin. The simultaneous use of two fluorescent dyes (PI and Hoechst 33342), of which the mechanisms of penetration into the cell and the fluorescence spectra are different, allowed the identification of four types of cells in the sample: live cells (weak, dull light blue fluorescence), early apoptotic cells (bright light blue fluorescence), late apoptotic cells (pink-purple fluorescence), and necrotic cells (intense red fluorescence).

THP1, HL60, and RPMI8226 cells were incubated with the test compounds for 4, 24, and 48 h. After an appropriate incubation time, cells were removed from the culture dishes, centrifuged, and suspended at a final concentration of 1 × 10^5^ cells in 1 mL of PBS. The fluorescent dyes Hoechst 33342 (0.13 mM) and PI (0.23 mM) were added and incubated for 10 min in the dark. The cell suspension was placed on a microscope slide. At least 300 cells per sample were counted in triplicate under an Olympus fluorescence microscope using an NB filter. The percentage of the particular cell types was determined from the total number of cells.

### 4.7. Visualization of Changes in Cell Membrane Integrity and Externalization of Phosphatidylserine 

Visualization of cells stained with annexin V–FITC and PI was applied according to the protocol of the manufacturer, BioVision Incorporated (annexin V–FITC apoptosis kit). This method is a useful tool for distinguishing live cells (not stained with any fluorochrome) from early apoptotic cells (stained with annexin V–FITC only), late apoptotic cells (stained with annexin V–FITC and PI), and necrotic cells (stained with PI only).

Briefly, cells were seeded at a density of 2.5 × 10^5^ cells/well and treated with the test compounds for 24 h. The cells were then washed with PBS and resuspended in 300 μL binding buffer. Then, 3 μL annexin V–FITC and 3 μL PI was added. The mixture was incubated for 10 min on ice. Samples were centrifuged, applied to chamber slides, and then visualized using fluorescence microscopy (Olympus IX70, Tokyo, Japan). Magnification was 200×.

### 4.8. Activity of Cysteine Proteases (Caspases)

The activation of executive (caspases 3/7) and initiating caspases (involved in the activation of the external (caspase 8) and internal (caspase 9) pathways) is widely known as a reliable indicator of cell apoptosis. DEVD–ProRed™, IETD–R110, and LEHD–AMC were used as fluorogenic markers for the activity of caspases 3/7, 8, and 9, respectively. Following caspase cleavage, the DEVD–ProRed, IETD–R110, and LEHD–AMC caspase substrates generate three different fluorophores, ProRed™ (red fluorescence), R110 (green fluorescence), and AMC (blue fluorescence), which can be easily monitored using flow cytometry.

THP1, HL60, and RPMI8226 cells were seeded in plates (1 × 10^6^), and then the compounds were added at the appropriate concentration. After an appropriate incubation time (4, 24, or 48 h), cells were transferred to cytometric tubes, resuspended in a prepared working solution containing the appropriate substrate (50 µL of substrate into 10 mL assay buffer), and incubated for 30 to 60 min at room temperature, protected from light. Cells were analyzed using a flow cytometer (Becton Dickinson, San Jose, CA, USA) using the following wavelengths: caspase 3/7, ex: 488 nm, em: 620 nm; caspase 8, ex: 490 nm, em: 525 nm; caspase 9, ex: 355 nm, em: 470 nm. The minimum number of events recorded in the sample was 20,000. The results are presented as the median fluorescence in the corresponding channel: PE–Texas Red, FITC, DAPI as % relative to control.

### 4.9. Mitochondrial Membrane Potential (Δψm)

Cells collected from the culture vessel (15 × 10^6^) were centrifuged (10 min, 1200 rpm), and the culture medium with phenol red was discarded. PBS was added to the pellet, and cells were centrifuged. In the next step, cells were suspended in a working culture medium without phenol red with JC–1 (10 µM) and incubated at 37 °C for 30 min in the dark. The cells were then centrifuged and washed with PBS to remove the dye, which otherwise could have adsorbed on the microplate well plastic and distorted the measurements. Cells prepared in this way were seeded into 96-well microplates at 8 × 10^4^ cells per well. The cells were incubated with the test compounds or CCCP, a mitochondrial uncoupling agent (20 and 100 µM), for 4–24 h. At the end of the treatment, the fluorescence of both JC–1 monomers and dimers was measured on a Fluoroskan Ascent FL microplate reader using filter pairs of 530/590 nm (dimers) and 485/538 nm (monomers). The results shown in the figures are expressed as a ratio of dimer fluorescence to monomer fluorescence in relation to the control fluorescence ratio, taken as 100%. The cells presented in the images were incubated with drugs for 48 h. JC–1 fluorescence was photographed immediately after drug treatment using an inverted Olympus IX70 fluorescence microscope (Olympus, Tokyo, Japan).

### 4.10. Measurement of DNA Damage (the Alkaline Version of the Comet Assay)

Single-cell agarose gel electrophoresis, commonly referred to as the comet assay, is used to detect alkyl labile sites, as well as single- and double-strand breaks in DNA induced by genotoxic agents. The method involves electrophoretic separation of nuclear DNA so that DNA fragmentation can be observed. The method was performed according to the protocol described in our previous articles [7,13,46].

The cells were plated in 12-well plates (2.5 × 10^4^ cells) and treated with MEL, EM–I–MEL, and EM–T–MEL at the indicated concentrations for 4, 24, and 48 h at 37 °C. The cells were washed with PBS, and the pellet was resuspended in 50 μL of 0.75% low-melting point (LMP) agarose in PBS, pH 7.4. The samples were applied to heated primary slides that had previously been coated with 1% normal-melting point (NMP) agarose. Slides prepared in this manner were then subjected to alkaline lysis (2.5 M NaCl, 100 mM EDTA, 10 mM Tris, 1% Triton X-100, pH 9.0) for a minimum of 1 h at 4 °C. Slides were then placed in developing buffer (300 mM NaOH, 1 mM EDTA) for 20 min and then in electrophoresis buffer (30 mM NaOH, 1 mM EDTA). Electrophoresis was conducted at 29 V and 30 mA for 20 min. Slides were then stained with DAPI (2 μg/mL) in the dark. Fifty randomly selected cells from each slide were analyzed using an Eclipse fluorescence microscope (Nikon, Japan) attached to a COHU 4910 video camera (Cohu, Inc., San Diego, CA, USA) equipped with a UV–1A filter block and connected to the Lucia-Comet v. 6.0 image analysis system (Laboratory Imaging, Prague, Czech Republic). The percentage of DNA in the comet tail is an indicator of DNA damage.

### 4.11. Statistical Analysis

Data are presented as the mean ± standard deviation (SD). Analysis of variance (ANOVA) with Tukey’s post hoc test was used for multiple comparisons. All statistics were calculated using STATISTICA (StatSoft, Tulsa, OK, USA). A *p*-value < 0.05 was considered significant. All figures include descriptions of statistically significant changes: * *p* < 0.05 statistically significant difference compared with control cells, # *p* < 0.05 statistically significant difference between samples incubated with melphalan and melphalan derivatives.

## 5. Conclusions

Research in cancer biology has led to the elucidation of the mechanism of action of anticancer agents. It has also provided a basis for the successful design of new drugs. The development of new analogs of anticancer drugs is a complicated task. Applying chemical principles and analyzing chemical structures together with biological activity enables the synthesis of derivatives with higher biological properties than the parent drug. In vitro studies have confirmed the hypothesis that chemical modification of melphalan leads to significantly higher cytotoxic and genotoxic activities. These modifications include esterifying the carboxyl group and replacing the amino group with amidine containing a thiomorpholine residue with two heteroatoms (N and S) in its structure. The generation of apoptotic bodies and reductions in the mitochondrial membrane potential suggest that these compounds induce apoptosis, the preferred type of cell death. 

**Figure 9:**
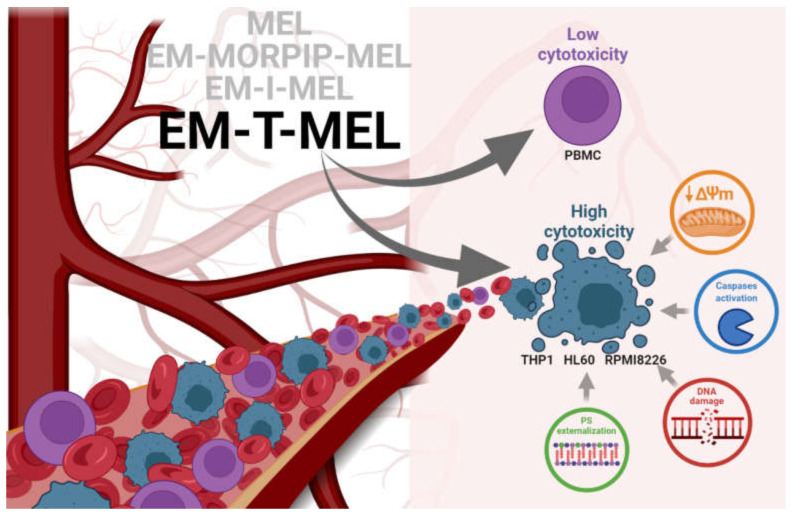
Proposed model of the molecular pathways of the new MEL derivatives with respect to neoplastic cells (THP1, HL60, and RPMI8226) and PBMC.

This studies allowed us to select EM–T–MEL as the most active analog. It also significantly deepened the knowledge on improving the biological activity of the currently used chemotherapeutic agent (Figure 9).

## Figures and Tables

**Table 1:** Druglikeness parameters estimated according to Lipinski’s and Veber’s rules. HBDs ^a^: number of hydrogen bond donors; HBAs ^b^: number of hydrogen bond acceptors; NBR ^c^: number of rotatable bonds; TPSA ^d^: total polar surface area.

	Lipinski’s Rules				Veber’s Rule	
Compound	MW ≤500 Da	LogP ≤5	HBDs ^a^ ≤5	HBAs ^b^ ≤10	NBR ^c^ ≤10	TPSA ^d^ ≤140
EM–I–MEL	448.39	4.46	0	3	11	45.14
EM–T–MEL	432.41	3.61	0	3	11	70.44
EM–MORPIP–MEL	499.47	3.36	0	5	12	57.61

## Data Availability

The datasets presented during in the current study are available from the corresponding author on reasonable request.

## Supplementary Materials

The following are available online at www.mdpi.com/article/10.3390/ijms23031760/s1.

## References

[1] Parra M., Baptista M.J., Genescà E., Llinàs-Arias P., Esteller M. (2020). Genetics and epigenetics of leukemia and lymphoma: From knowledge to applications, meeting report of the Josep Carreras Leukaemia Research Institute. Hematol. Oncol..

[2] Oriol A., Larocca A., Leleu X., Hajek R., Hassoun H., Rodríguez-Otero P., Paner A., Schjesvold F.H., Gullbo J., Richardson P.G. (2020). Melflufen for relapsed and refractory multiple myeloma. Expert Opin. Investig. Drugs.

[3] Rosiñol L., Oriol A., Rios R., Sureda A., Blanchard M.J., Hernández M.T., Martínez-Martínez R., Moraleda J.M., Jarque I., Bargay J. (2019). Bortezomib, lenalidomide, and dexamethasone as induction therapy prior to autologous transplant in multiple myeloma. Blood.

[4] Pinto V., Bergantim R., Caires H.R., Seca H., Guimarães J.E., Vasconcelos M.H. (2020). Multiple myeloma: Available therapies and causes of drug resistance. Cancers.

[5] Singh R.K., Kumar S., Prasad D.N., Bhardwaj T.R. (2018). Therapeutic journery of nitrogen mustard as alkylating anticancer agents: Historic to future perspectives. Eur. J. Med. Chem..

[6] Yang J., Terebelo H.R., Zonder J.A. (2012). Secondary Primary Malignancies in Multiple Myeloma: An Old Nemesis Revisited. Adv. Hematol..

[7] Poczta A., Rogalska A., Marczak A. (2021). Treatment of Multiple Myeloma and the Role of Melphalan in the Era of Modern Therapies—Current Research and Clinical Approaches. J. Clin. Med..

[8] Bergel F., Stock J.A. (1954). Cyto-active Amino-acid and Peptide Derivatives. J. Chem. Soc..

[9] Szekerke B.M., Wade R., Bergel F. (1968). Cyto-active Amino-acids and Peptides. Part XIV. Poly-and Copoly-(amino-acyl) derivatives of melphalan. J. Chem. Soc. C Organic.

[10] Everett J.L., Baker M.H., Bergel F. (1968). Cyto-active Amino-acids and Peptides. Part Xlll Fluorescent Derivatives of Melphalan t and other Amino-acids. J. Chem. Soc. C Organic.

[11] Gullbo J., Tullberg M., Våbenø J., Ehrsson H., Lewensohn R., Nygren P., Larsson R., Luthman K. (2003). Structure-Activity Relationship for Alkylating Dipeptide Nitrogen Mustard Derivatives. Oncol. Res. Featur. Preclin. Clin. Cancer Ther..

[12] Lu B., Huang D., Zheng H., Huang Z., Xu P., Xu H., Yin Y., Liu X., Li D., Zhang X. (2013). Preparation, characterization, and in vitro efficacy of O-carboxymethyl chitosan conjugate of melphalan. Carbohydr. Polym..

[13] Gajek A., Poczta A., Łukawska M., Cecuda-Adamczewska V., Tobiasz J., Marczak A. (2020). Chemical modification of melphalan as a key to improving treatment of haematological malignancies. Sci. Rep..

[14] Małkiewicz M.A., Szarmach A., Sabisz A., Cubała W.J., Szurowska E., Winklewski P.J. (2019). Blood-brain barrier permeability and physical exercise. J. Neuroinflammation.

[15] Al-Ghorbani M., Bushra B.A., Zabiulla S.V.M., Mamatha S., Khanum S.A. (2015). Piperazine and morpholine: Synthetic preview and pharmaceutical applications. Res. J. Pharm. Technol..

[16] Al-Ghorbani M., Vigneshwaran V., Ranganatha V.L., Prabhakar B.T., Khanum S.A. (2015). Synthesis of oxadiazole-morpholine derivatives and manifestation of the repressed CD31 Microvessel Density (MVD) as tumoral angiogenic parameters in Dalton’s Lymphoma. Bioorganic Chem..

[17] Lenci E., Calugi L., Trabocchi A. (2021). Occurrence of Morpholine in Central Nervous System Drug Discovery. ACS Chem. Neurosci..

[18] Arshad F., Khan M.F., Akhtar W., Alam M.M., Nainwal L.M., Kaushik S.K., Akhter M., Parvez S., Hasan S.M., Shaquiquzzaman M. (2019). Revealing quinquennial anticancer journey of morpholine: A SAR based review. Eur. J. Med. Chem..

[19] Hamid R., Rotshteyn Y., Rabadi L., Parikh R., Bullock P. (2004). Comparison of alamar blue and MTT assays for high through-put screening. Toxicol. Vitr..

[20] Van Kan M., Burns K.E., Browett P., Helsby N.A. (2019). A higher throughput assay for quantification of melphalan-induced DNA damage in peripheral blood mononuclear cells. Sci. Rep..

[21] Bulumulla C., Kularatne R.N., Catchpole T., Takacs A., Christie A., Gilfoyle A., Nguyen T.D., Stefan M.C., Csaky K.G. (2020). Investigating the effect of esterification on retinal pigment epithelial uptake using rhodamine B derivatives. Transl. Vis. Sci. Technol..

[22] Wang C.-L., Guo C., Zhou Y., Wang R. (2009). In vitro and in vivo characterization of opioid activities of C-terminal esterified endomorphin-2 analogs. Peptides.

[23] De Oliveira D.P., Moreira T.D.V., Batista N.V., Filho J.D.D.S., Amaral F.A., Teixeira M.M., de Pádua R.M., Braga F.C. (2018). Esterification of trans-aconitic acid improves its anti-inflammatory activity in LPS-induced acute arthritis. Biomed. Pharmacother..

[24] Chvapil M., Kielar F., Liska F., Silhankova A., Brendel K. (2005). Synthesis and evaluation of long-acting D-penicillamine derivatives. Connect. Tissue Res..

[25] Bielawska A., Bielawski K., Anchim T. (2007). Amidine analogues of melphalan: Synthesis, cytotoxic activity, and DNA binding properties. Arch. Der Pharm..

[26] Bielawski K., Bielawska A., Sosnowska K., Miltyk W., Winnicka K., Pałka J. (2006). Novel amidine analogue of melphalan as a specific multifunctional inhibitor of growth and metabolism of human breast cancer cells. Biochem. Pharmacol..

[27] Zhao Y., Wang L., Pan J. (2015). The role of L-type amino acid transporter 1 in human tumors. Intractable Rare Dis. Res..

[28] Hayashi K., Jutabha P., Endou H., Sagara H., Anzai N. (2013). LAT1 Is a Critical Transporter of Essential Amino Acids for Immune Reactions in Activated Human T Cells. J. Immunol..

[29] Mynott R.L., Wallington-Beddoe C.T. (2021). Drug and Solute Transporters in Mediating Resistance to Novel Therapeutics in Multiple Myeloma. ACS Pharmacol. Transl. Sci..

[30] Pegoraro A., Orioli E., De Marchi E., Salvestrini V., Milani A., Di Virgilio F., Curti A., Adinolfi E. (2020). Differential sensitivity of acute myeloid leukemia cells to daunorubicin depends on P2X7A versus P2X7B receptor expression. Cell Death Dis..

[31] Lee C.-K., Wang S., Huang X., Ryder J., Liu B. (2010). HDAC inhibition synergistically enhances alkylator-induced DNA damage responses and apoptosis in multiple myeloma cells. Cancer Lett..

[32] Cordelli E., Cinelli S., Lascialfari A., Ranaldi R., Pacchierotti F. (2004). Melphalan-induced DNA damage in p53(+/−) and wild type mice analysed by the comet assay. Mutat. Res..

[33] Xu H., He J., Zhang Y., Fan L., Zhao Y., Xu T., Nie Z., Li X., Huang Z., Lu B. (2015). Synthesis and in vitro evaluation of a hyaluronic acid-quantum dots-melphalan conjugate. Carbohydr. Polym..

[34] Keoni C.L.I., Brown T.L. (2015). Inhibition of apoptosis and efficacy of pan caspase inhibitor, Q-VD-OPh, in models of human disease. J. Cell Death.

[35] Verrax J., Dejeans N., Sid B., Glorieux C., Calderon P.B. (2011). Intracellular ATP levels determine cell death fate of cancer cells exposed to both standard and redox chemotherapeutic agents. Biochem. Pharmacol..

[36] El Dein O.S., Gallerne C., Brenner C., Lemaire C. (2012). Increased expression of VDAC1 sensitizes carcinoma cells to apoptosis induced by DNA cross-linking agents. Biochem. Pharmacol..

[37] Matsura T., Kai M., Jiang J., Babu H., Kini V., Kusumoto C., Yamada K., Kagan V.E. (2004). Endogenously Generated Hydrogen Peroxide Is Required for Execution of Melphalan-Induced Apoptosis as Well as Oxidation and Externalization of Phosphatidylserine. Chem. Res. Toxicol..

[38] Xu X., Lai Y., Hua Z.-C. (2019). Apoptosis and apoptotic body: Disease message and therapeutic target potentials. Biosci. Rep..

[39] Broker L.E., Kruyt F.A.E., Giaccone G. (2005). Cell death independent of caspases: A review. Clin. Cancer Res..

[40] Vitale I., Manic G., Castedo M., Kroemer G. (2017). Caspase 2 in mitotic catastrophe: The terminator of aneuploid and tetraploid cells. Mol. Cell. Oncol..

[41] Čáňová K., Rozkydalová L., Vokurková D., Rudolf E. (2018). Flubendazole induces mitotic catastrophe and apoptosis in melanoma cells. Toxicol. Vitr..

[42] Mc Gee M.M. (2015). Targeting the Mitotic Catastrophe Signaling Pathway in Cancer. Mediat. Inflamm..

[43] Erenpreisa J., Kalejs M., Ianzini F., Kosmacek E., Mackey M., Emzinsh D., Cragg M., Ivanov A., Illidge T. (2005). Segregation of genomes in polyploid tumour cells following mitotic catastrophe. Cell Biol. Int..

[44] Cives M., Ciavarella S., Rizzo F.M., De Matteo M., Dammacco F., Silvestris F. (2013). Bendamustine overcomes resistance to melphalan in myeloma cell lines by inducing cell death through mitotic catastrophe. Cell. Signal..

[45] Daina A., Michielin O., Zoete V. (2017). SwissADME: A free web tool to evaluate pharmacokinetics, drug-likeness and medicinal chemistry friendliness of small molecules. Sci. Rep..

[46] Gajek A., Rogalska A., Koceva-Chyła A. (2019). Aclarubicin in subtoxic doses reduces doxorubicin cytotoxicity in human non-small cell lung adenocarcinoma (A549) and human hepatocellular carcinoma (HepG2) cells by decreasing DNA damage. Toxicol. Vitr..

