# Supplementary material for: Synthesis and In Vitro Activity of Novel Melphalan Analogs in Hematological Malignancy Cells
Source: Int J Mol Sci. 2022 Feb 3;23(3):1760. doi: 10.3390/ijms23031760 (PMC8836188; doi:10.3390/ijms23031760)
Supplement: Supplementary file 1 [file ijms-23-01760-s001.zip › ijms-1541326-supplementary.pdf]

Suplementary materials

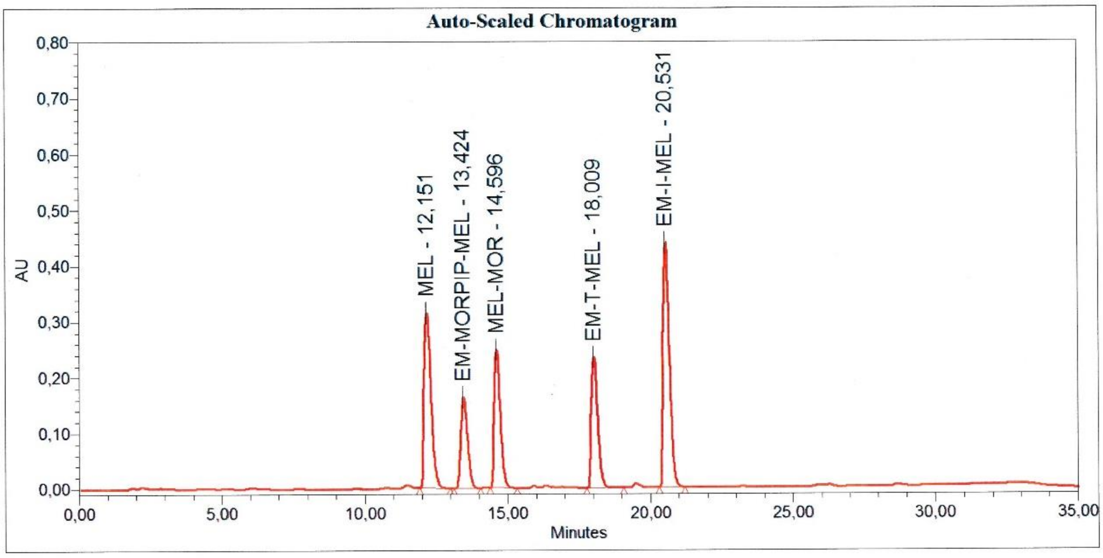

Figure S1. HPLC Chromatogram of MEL, MOR–MEL and new derivatives described in this paper.

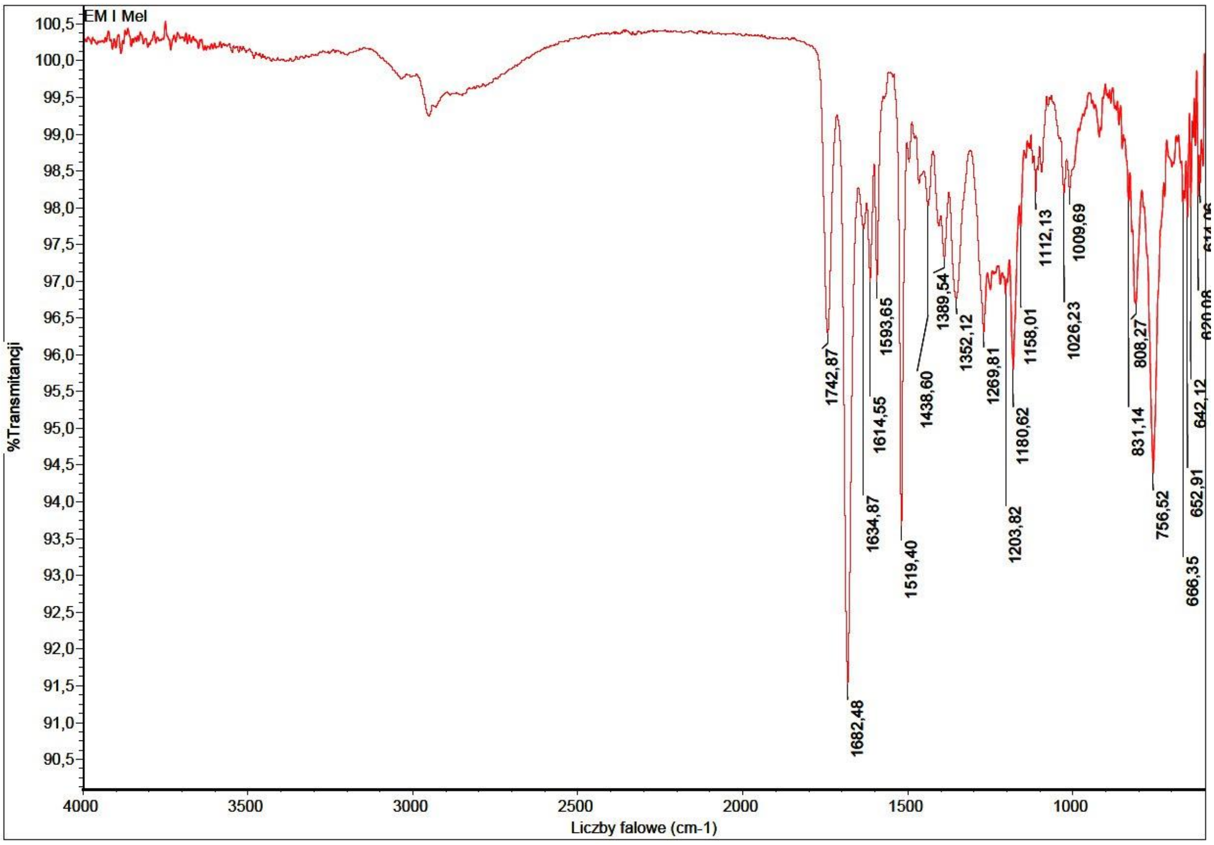

Figure S2. IR spectrum of the EM–I–MEL.

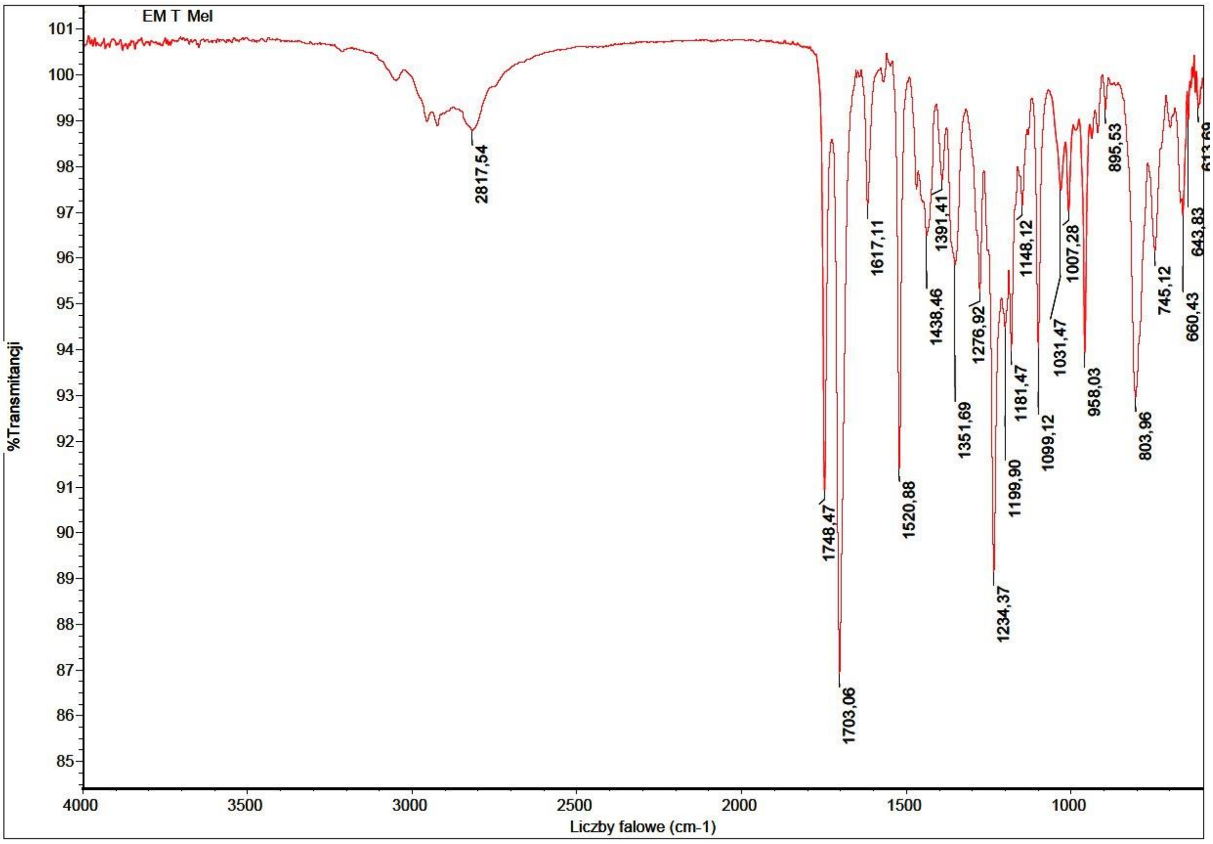

Figure S3. IR spectrum of the EM–T–MEL.

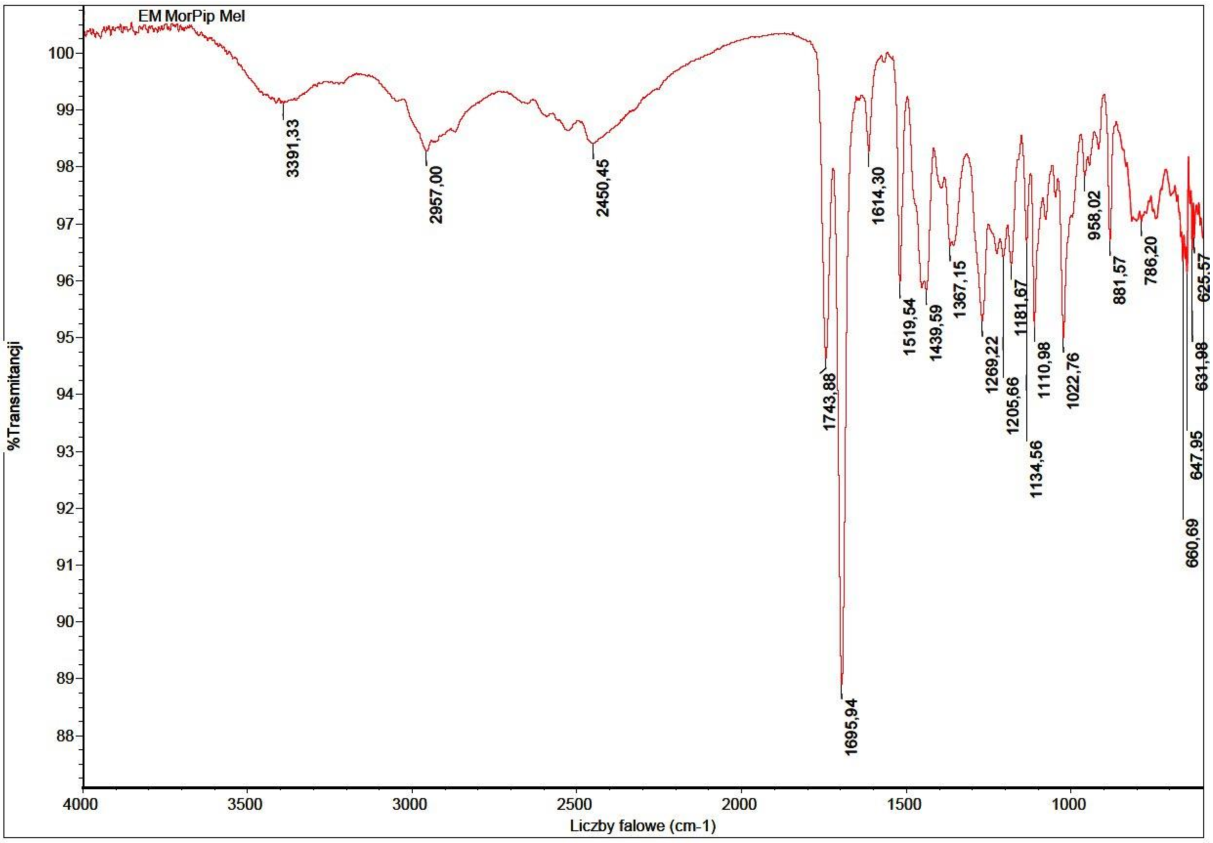

Figure S4. IR spectrum of the EM–MORPIP–MEL.

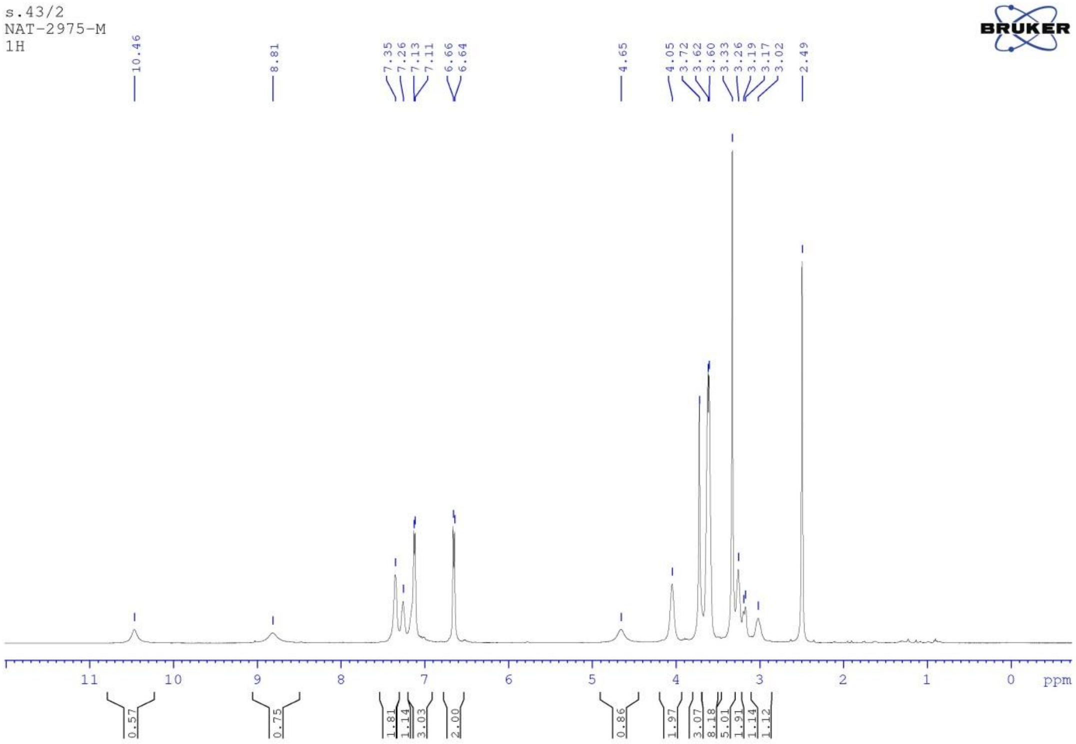

Figure S5. ^{1}H NMR spectrum of the EM–I–MEL.

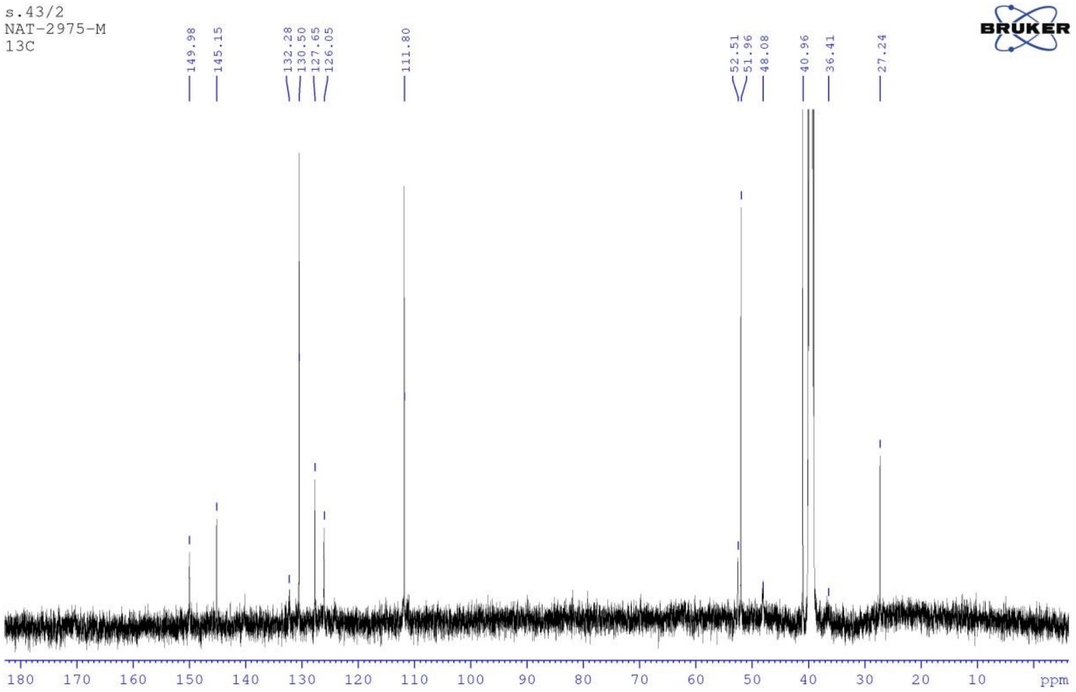

Figure S6. ^{13}C NMR spectrum of the EM–I–MEL.

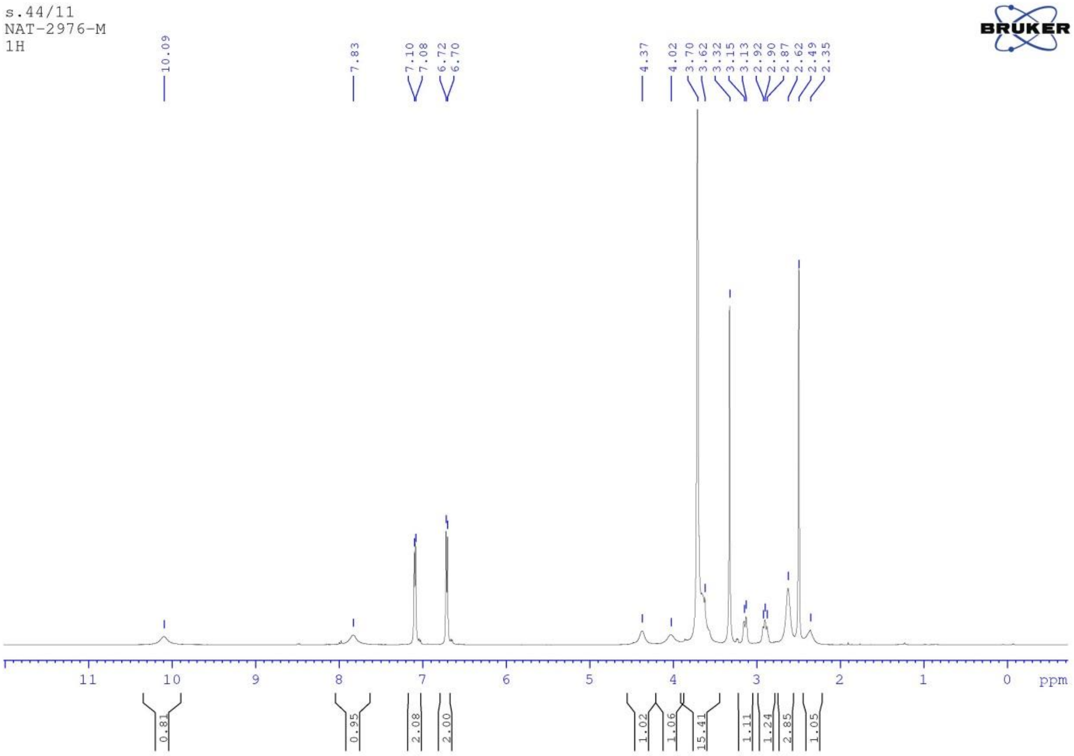

Figure S7. ^{1}H NMR spectrum of the EM–T–MEL.

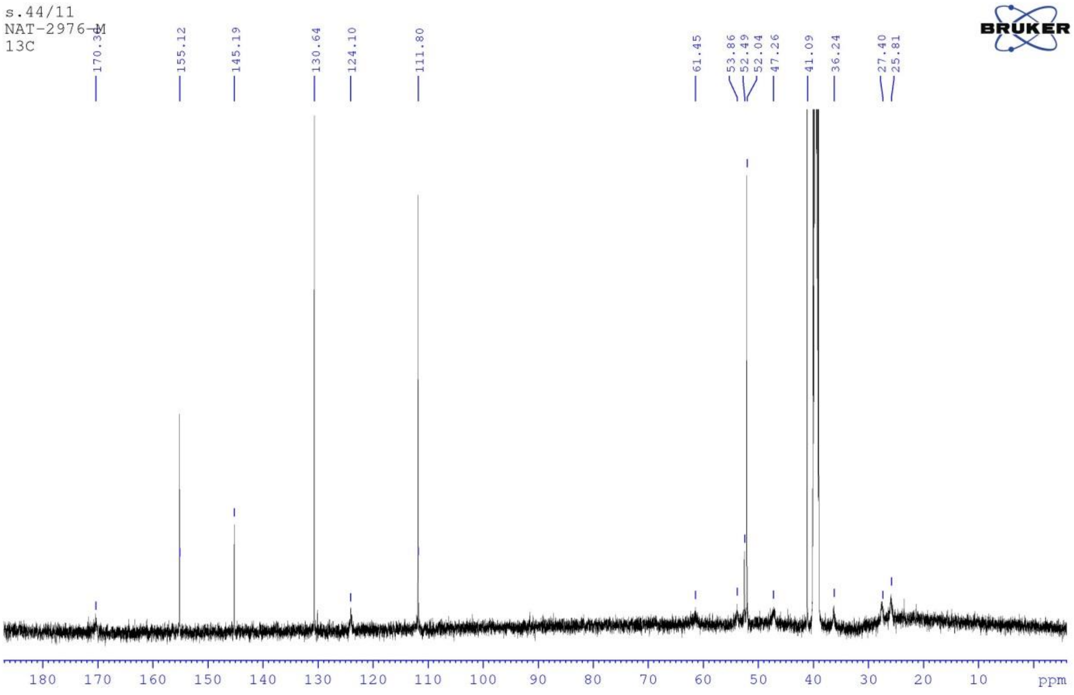

Figure S8. ^{13}C NMR spectrum of the EM–T–MEL.

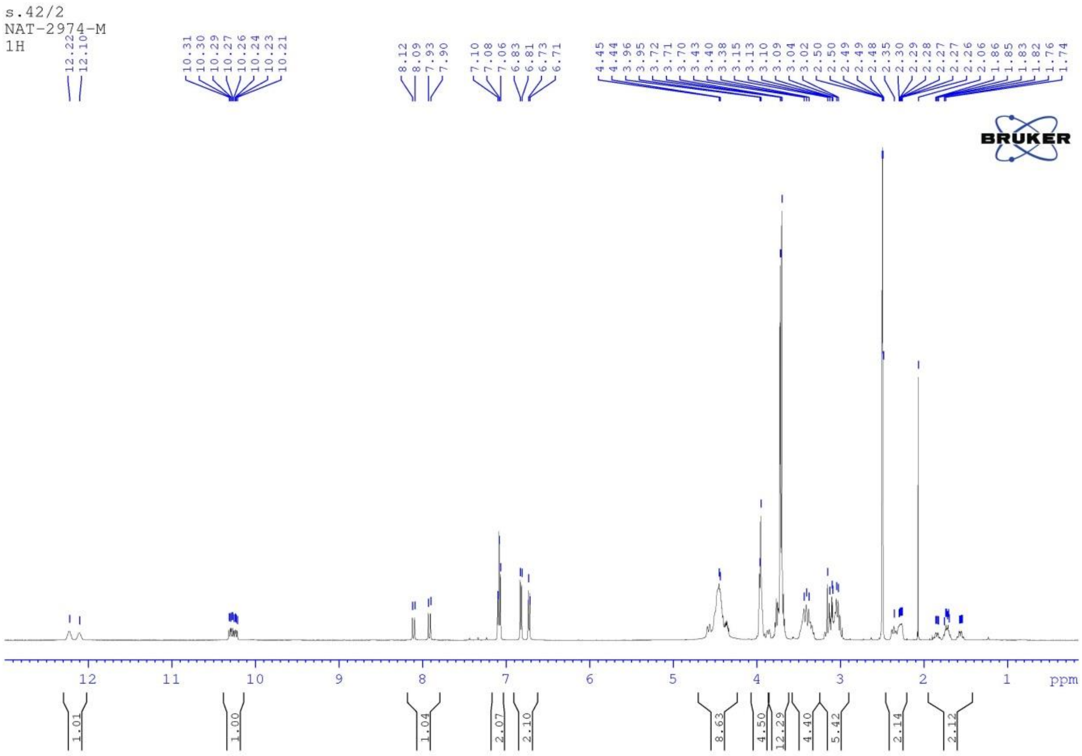

Figure S9. ^{1}H NMR spectrum of the EM–MORPIP–MEL.

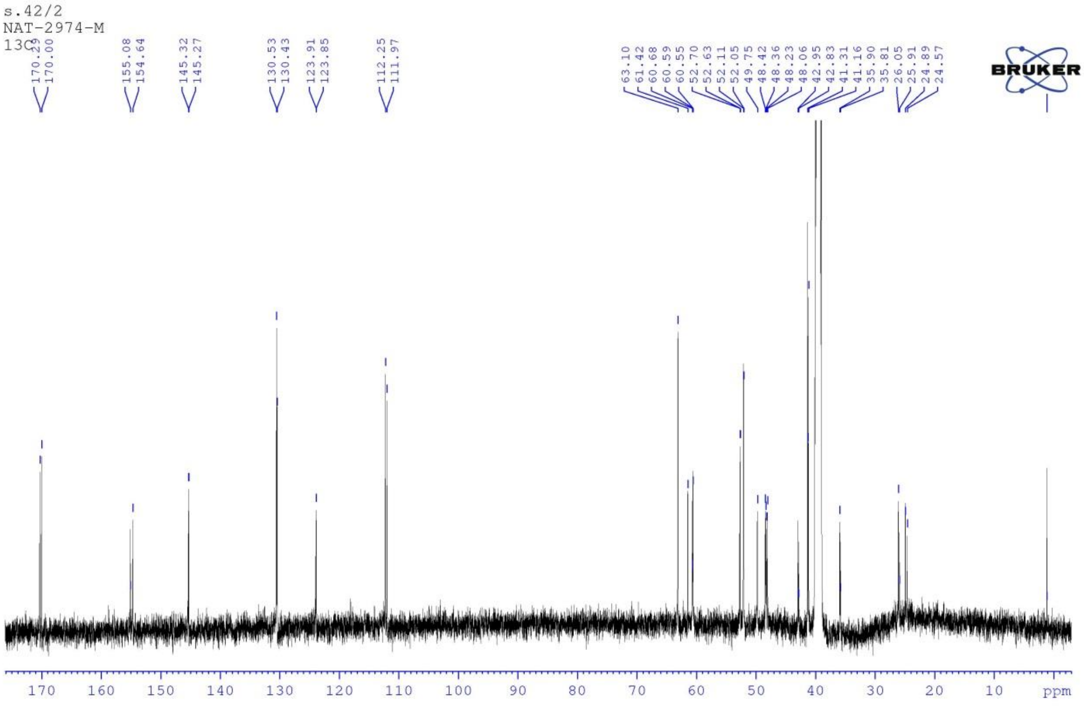

Figure S10. ^{13}C NMR spectrum of the EM–MORPIP–MEL.

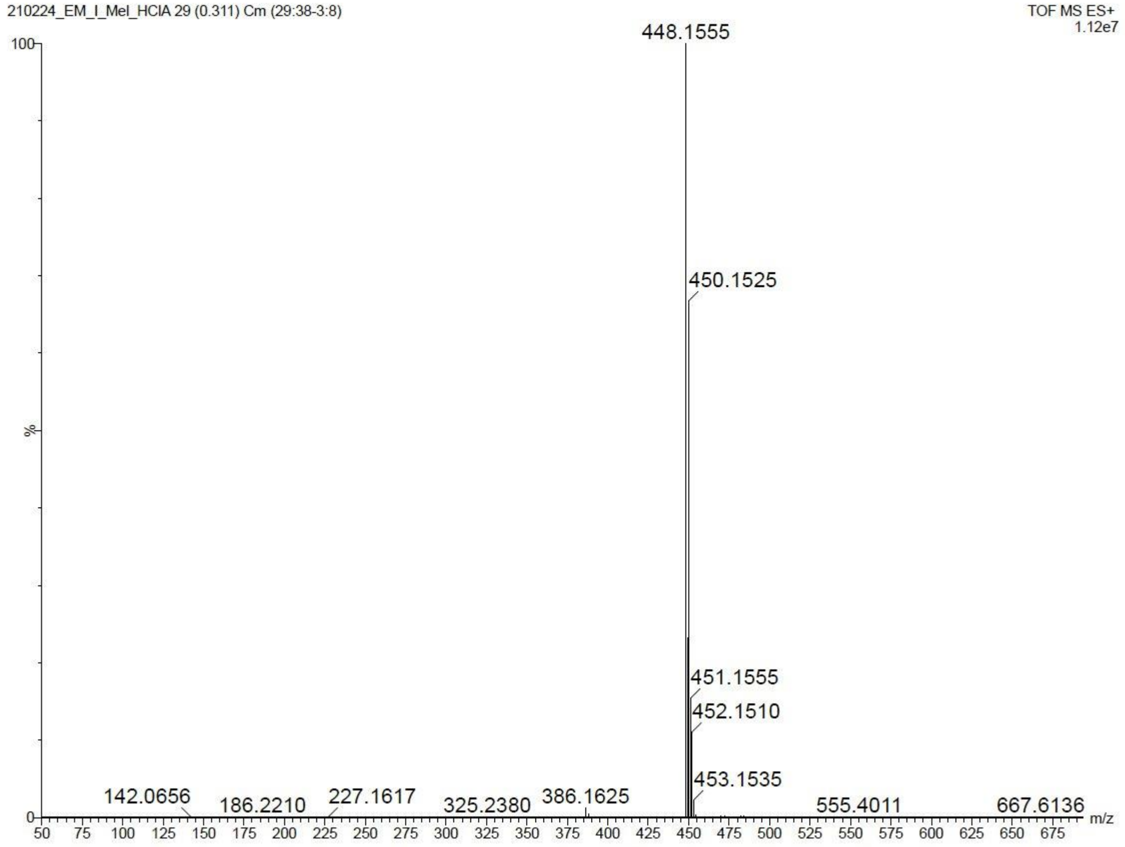

Figure S11. HRMS spectrum of the EM–I–MEL.

Elemental Composition Report **Page 1**

Single Mass Analysis
Tolerance = 5.0 PPM / DBE: min = -1.5, max = 80.0
Element prediction: Off
Number of isotope peaks used for i-FIT = 9

Monoisotopic Mass, Even Electron Ions
404 formula(e) evaluated with 2 results within limits (all results (up to 1000) for each mass)
Elements Used:
C: 0-50 H: 0-80 N: 0-5 O: 0-6 Cl: 1-2

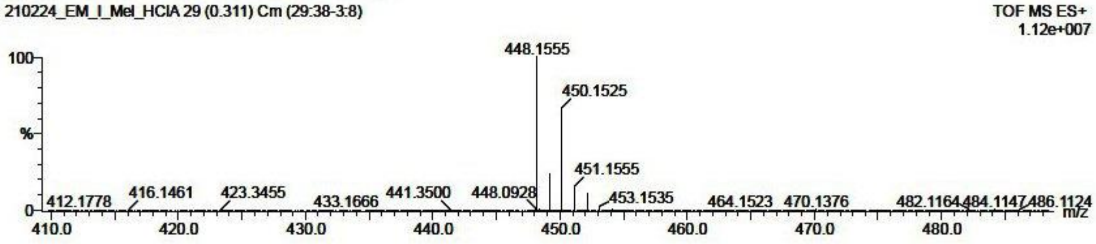

Minimum:				-1.5				
Maximum:		5.0	5.0	80.0				
Mass	Calc. Mass	mDa	PPM	DBE	i-FIT	Norm	Conf(%)	Formula
448.1555	448.1559	-0.4	-0.9	10.5	1625.1	0.000	100.00	C23 H28 N3 O2 Cl2
	448.1540	1.5	3.3	15.5	1646.4	21.272	0.00	C24 H23 N5 O2 Cl

Figure S12. Elemental analysis of the EM–I–MEL.

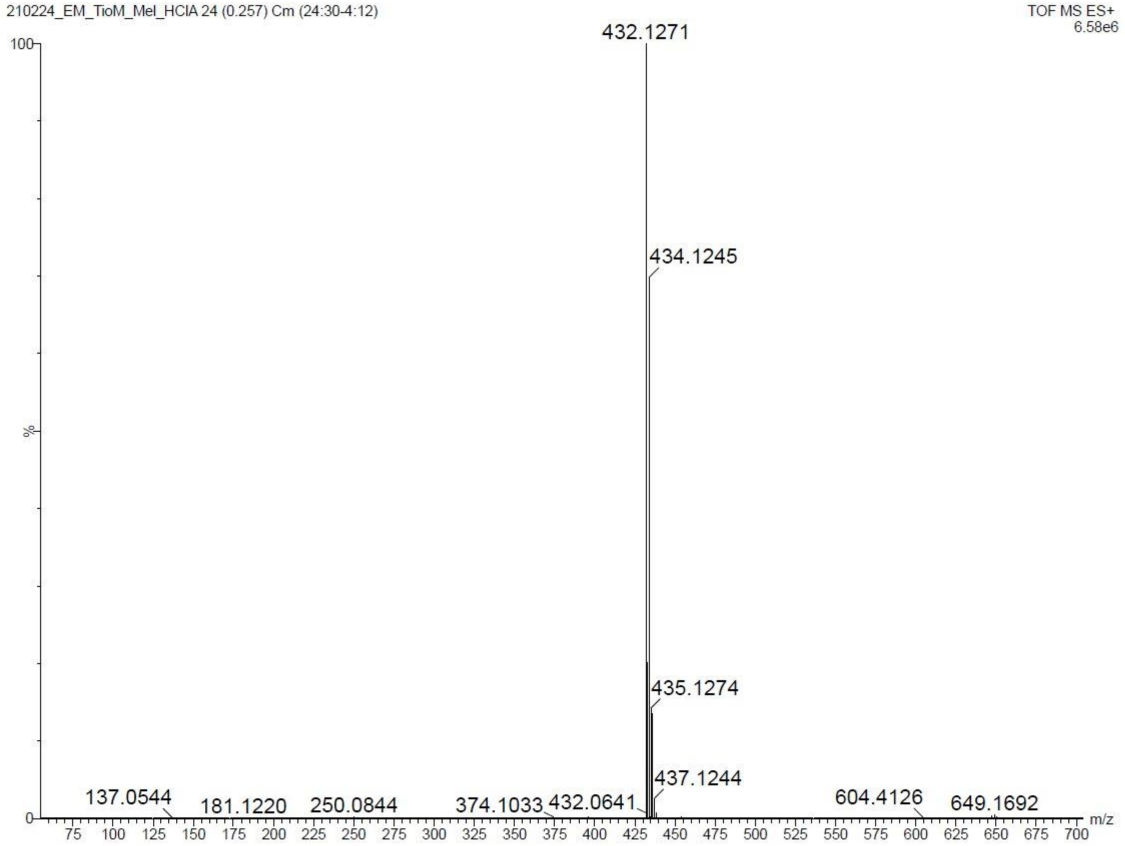

Figure S13. HRMS spectrum of the EM–T–MEL.

Elemental Composition Report

Page 1

Single Mass Analysis

Tolerance = 5.0 PPM / DBE: min = -1.5, max = 80.0
Element prediction: Off
Number of isotope peaks used for i-FIT = 9

Monoisotopic Mass, Even Electron Ions
356 formula(e) evaluated with 2 results within limits (all results (up to 1000) for each mass)
Elements Used:
C: 0-50 H: 0-80 N: 0-5 O: 0-6 S: 1-1 Cl: 1-2

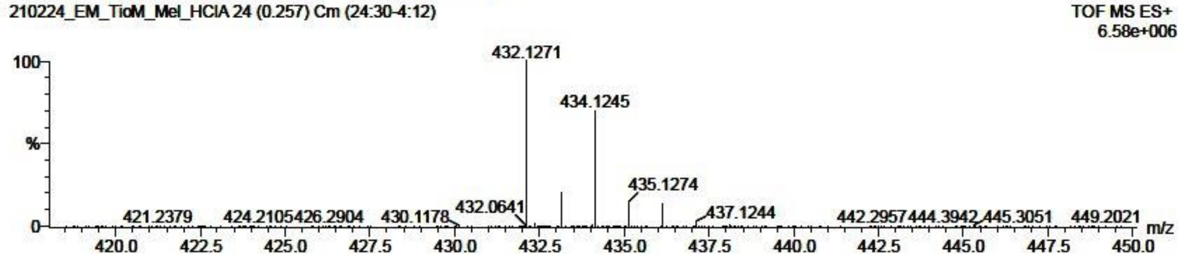

Mass	Calc. Mass	mDa	PPM	DBE	i-FIT	Norm	Conf(%)	Formula
Minimum:				-1.5				
Maximum:		5.0	5.0	80.0				
432.1271	432.1279	-0.8	-1.9	6.5	1080.8	0.000	100.00	C19 H28 N3 O2 S Cl2
	432.1261	1.0	2.3	11.5	1108.6	27.788	0.00	C20 H23 N5 O2 S Cl

Figure S14. Elemental analysis of the EM–T–MEL.

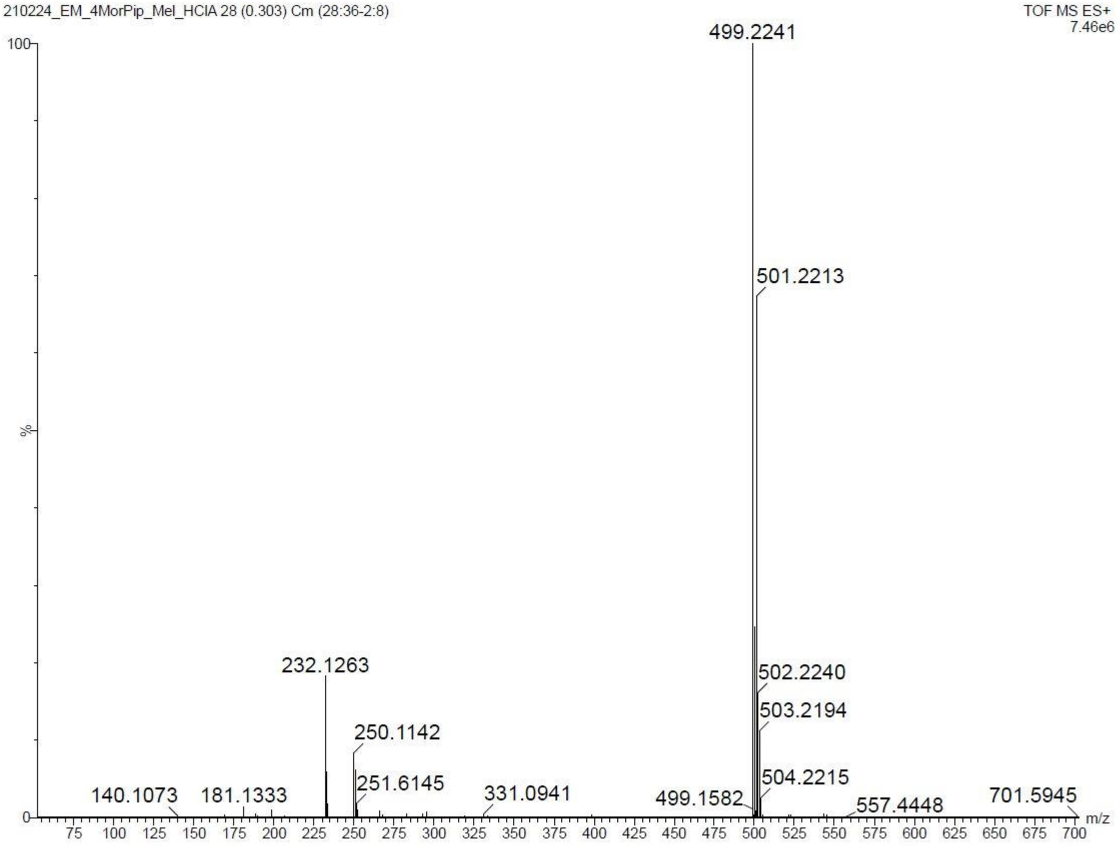

Figure S15. HRMS spectrum of the EM–MORPIP–MEL.

Elemental Composition Report **Page 1**

Single Mass Analysis
Tolerance = 5.0 PPM / DBE: min = -1.5, max = 80.0
Element prediction: Off
Number of isotope peaks used for i-FIT = 9

Monoisotopic Mass, Even Electron Ions
455 formula(e) evaluated with 3 results within limits (all results (up to 1000) for each mass)
Elements Used:
C: 0-50 H: 0-80 N: 0-5 O: 0-6 Cl: 1-2

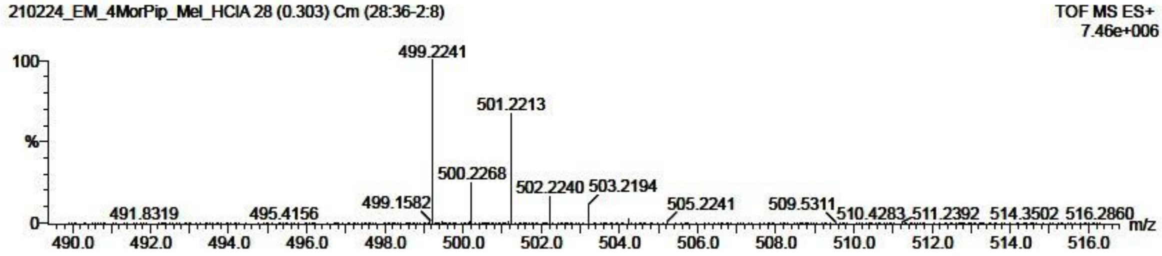

Minimum:				-1.5				
Maximum:		5.0	5.0	80.0				
Mass	Calc. Mass	mDa	PPM	DBE	i-FIT	Norm	Conf(%)	Formula
499.2241	499.2243	-0.2	-0.4	7.5	1271.1	0.000	100.00	C24 H37 N4 O3 Cl2
	499.2251	-1.0	-2.0	11.5	1288.2	17.078	0.00	C29 H36 O5 Cl
	499.2265	-2.4	-4.8	16.5	1288.7	17.583	0.00	C30 H32 N4 O Cl

Figure S16. Elemental analysis of the EM–MORPIP–MEL.
